# Clinical Outcomes of Pulsed Field Vs. Thermal Ablation for Paroxysmal Atrial Fibrillation: Systematic Review and Meta‐Analysis of Randomized Evidence

**DOI:** 10.1002/joa3.70446

**Published:** 2026-08-20

**Authors:** Asad Ali Ahmed Cheema, Reshail Asif, Unsa Arif, Manahil Malik, Sarah Misbah, Eraj Nadeem, Muhammad Iftikhar Alam, Deep Birjani, Syed Umair Ahmed, FNU Hamna, Hadia Haseeb Ahmad, Fizza Sajid, Shahrukh Nadeem, Zaitoon Hanif, Abu Huraira Bin Gulzar

**Affiliations:** ^1^ International School of Medicine International University of Kyrgyzstan Bishkek Kyrgyzstan; ^2^ Dow Medical College Karachi Pakistan; ^3^ Continental Medical College Lahore Punjab Pakistan; ^4^ Women Medical College Abbottabad Pakistan; ^5^ Texas Health Resources HEB/Denton Denton Texas USA; ^6^ Liaquat National Medical College Karachi Pakistan; ^7^ Ministry of Health Kuwait City Kuwait; ^8^ Dow University of Health Sciences Karachi Pakistan; ^9^ Bolan Medical College Quetta Pakistan; ^10^ Fatima Jinnah Medical University Lahore Pakistan; ^11^ Ivane Javakhishvili Tbilisi State University Tbilisi Georgia; ^12^ King Edward Medical University Lahore Pakistan; ^13^ Rutgers New Jersey Medical School/Trinitas Regional Medical Center Newark and Elizabeth New Jersey USA; ^14^ Jinnah Sindh Medical University Karachi Pakistan; ^15^ Services Institute of Medical Sciences Lahore Pakistan

**Keywords:** atrial fibrillation, catheter ablation, meta‐analysis, pulsed field ablation, thermal ablation

## Abstract

**Aims:**

Pulsed field ablation (PFA) is a non‐thermal alternative to conventional thermal ablation for paroxysmal atrial fibrillation (AF). We conducted an updated systematic review and meta‐analysis of randomized evidence comparing efficacy, safety, and procedural outcomes.

**Methods:**

PubMed/MEDLINE and the Cochrane Central Register of Controlled Trials were searched from inception through March 2026 for randomized controlled trials (RCTs) comparing PFA with thermal ablation in adults undergoing first catheter ablation for paroxysmal AF. Risk ratios (RRs) or mean differences (MDs) with 95% confidence intervals (CIs) were pooled using random‐effects models.

**Results:**

Four RCTs enrolling 1 393 patients were included. PFA showed no statistically detectable difference in 12‐month treatment success (RR, 1.04; 95% CI, 0.97 to 1.12; *I* ^2^ = 7%), recurrent atrial arrhythmias (RR, 1.00; 95% CI, 0.77 to 1.29; *I* ^2^ = 42%), or composite serious adverse events (RR, 0.71; 95% CI, 0.32 to 1.57; I^2^ = 7%). PFA shortened procedure time (MD, −22.42 min; 95% CI, −33.44 to −11.39; I^2^ = 91%) and LA dwell time (MD, −25.73 min; 95% CI, −34.66 to −16.79; *I* ^2^ = 92%), but increased fluoroscopy time (MD, 7.34 min; 95% CI, 0.39 to 14.30; *I* ^2^ = 98%).

**Conclusions:**

Across the included trial configurations, 12‐month efficacy estimates did not differ detectably. Sparse safety events and wide confidence intervals precluded equivalence claims. Pooled procedure and LA dwell times favored PFA, but platform, comparator, and workflow heterogeneity limit generalizability. Procedural findings are exploratory and should not be interpreted as direct energy‐specific effects.

## Graphical abstract

Across four randomized trials including 1 393 patients with paroxysmal atrial fibrillation, pulsed field ablation showed no statistically clear difference in 12‐month treatment success vs. thermal ablation. Safety estimates remained imprecise. Procedure and left atrial dwell times were shorter, whereas fluoroscopy time was longer, with substantial heterogeneity observed overall.

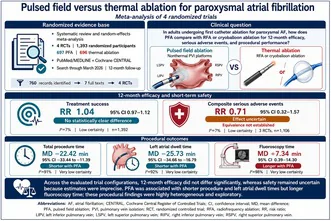

**Abbreviations**
AAD: antiarrhythmic drug
AECG: ambulatory electrocardiography
AF: atrial fibrillation
AFL: atrial flutter
AI: ablation index
AT: atrial tachycardia
BMI: body mass index
CBA: cryoballoon ablation
CENTRAL: Cochrane Central Register of Controlled Trials
CHA₂DS₂‐VASc: congestive heart failure, hypertension, age ≥ 75 years, diabetes mellitus, prior stroke/transient ischemic attack/thromboembolism, vascular disease, age 65–74 years, and sex category
CI: confidence interval
CT: computed tomography
CTI: cavotricuspid isthmus
DM: diabetes mellitus
ECG: electrocardiogram
FAS: full analysis set
GA: general anesthesia
GRADE: Grading of Recommendations Assessment, Development and Evaluation
HTN: hypertension
ICE: intracardiac echocardiography
IQR: interquartile range
LA: left atrial
LVEF: left ventricular ejection fraction
MD: mean difference
MeSH: Medical Subject Headings
MRI: magnetic resonance imaging
NR: not reported
nsPFA: nanosecond pulsed field ablation
PFA: pulsed field ablation
PICO: Population, Intervention, Comparator, and Outcomes
PRISMA: Preferred Reporting Items for Systematic Reviews and Meta‐Analyses
PV: pulmonary vein
PVI: pulmonary vein isolation
RCT: randomized controlled trial
RF: radiofrequency
RFA: radiofrequency ablation
RoB 2: revised Cochrane Risk of Bias tool for randomized trials
RR: risk ratio
SAE: serious adverse event
SD: standard deviation
SE: systemic embolism
TIA: transient ischemic attack

## 1 Introduction

Atrial fibrillation (AF) and atrial flutter (AFL) remain among the most common sustained cardiac arrhythmias worldwide, affecting an estimated 52.55 million people globally in 2021 [1]. Their clinical importance extends beyond symptoms because they are closely associated with stroke, heart failure, hospitalization, and death [2]. Because symptom burden and downstream cardiovascular risk often persist despite drug therapy, rhythm control remains a major component of contemporary AF management, and catheter ablation is now an established treatment option in appropriately selected symptomatic patients [2, 3]. The recognition that ectopic activity arising from the pulmonary veins can trigger AF established pulmonary vein isolation (PVI) as the mechanistic foundation of catheter‐based rhythm control [4]. In current practice, point‐by‐point radiofrequency ablation (RFA) and cryoballoon ablation (CBA) are the standard thermal approaches for achieving PVI, and randomized comparisons in paroxysmal AF have shown similar efficacy and safety between them [5].

Despite their established role, both RFA and CBA rely on thermal tissue injury. Durable lesion creation must therefore be balanced against collateral damage to adjacent structures and against procedural inefficiencies related to catheter manipulation and energy delivery [2, 3, 5]. Pulsed field ablation (PFA) has emerged as a non‐thermal alternative that produces irreversible electroporation with relative myocardial selectivity [6, 7]. Preclinical studies and early clinical investigations showed that PFA can create durable atrial lesions while limiting injury to surrounding tissues, particularly the esophagus, and subsequent multicenter and post‐approval experience reported favorable procedural performance with a low overall rate of major complications in contemporary practice [6, 7, 8, 9]. These observations created a strong rationale for direct randomized comparison of PFA with established thermal ablation strategies in paroxysmal AF.

Randomized comparator evidence has now expanded substantially. ADVENT demonstrated non‐inferior efficacy and safety of PFA compared with conventional thermal ablation at one year [10]. SINGLE SHOT CHAMPION extended this evidence against cryoballoon ablation by incorporating continuous implantable cardiac monitoring for recurrence detection [11]. More recently, BEAT PAROX‐AF and InsightPFA broadened the randomized evidence base further by evaluating PFA against optimized radiofrequency strategies and by introducing comparative data from distinct PFA platforms, including nanosecond technology [12, 13]. Earlier meta‐analyses by de Campos et al. and Qamar et al. examined broader questions using predominantly observational or nonrandomized evidence across heterogeneous atrial fibrillation populations, with de Campos et al. comparing PFA with thermal ablation and Qamar et al. primarily pooling single‐arm PFA outcomes [14, 15]. Amin et al. similarly combined one randomized trial with 16 nonrandomized studies, included both paroxysmal and persistent atrial fibrillation, and pooled radiofrequency ablation and cryoballoon ablation within a single thermal comparator framework. Esteves et al. focused specifically on pulsed field ablation vs. radiofrequency ablation in paroxysmal atrial fibrillation but included five observational studies alongside one randomized trial [16, 17]. Krishan et al. subsequently performed an RCT‐only meta‐analysis limited to ADVENT and SINGLE SHOT CHAMPION, whereas Mojica et al. included four RCTs spanning paroxysmal and persistent atrial fibrillation, including a dual‐energy pulsed field and radiofrequency ablation trial and a 24‐h physiology‐focused study, resulting in greater clinical and methodological heterogeneity [18, 19]. To address these limitations, we performed an updated meta‐analysis restricted to randomized trials specifically enrolling patients undergoing first catheter ablation for paroxysmal atrial fibrillation. We evaluated efficacy, safety, and procedural outcomes while explicitly accounting for differences in endpoint definitions, rhythm‐monitoring strategies, pulsed field ablation platforms, comparator modalities, and procedural workflows [10, 11, 12, 13, 14, 15, 16, 17, 18, 19].

## 2 Methods

### 2.1 Study Design and Reporting

This systematic review and meta‐analysis was conducted in accordance with the Preferred Reporting Items for Systematic Reviews and Meta‐Analyses 2020 (PRISMA 2020) statement and the methodological recommendations of the Cochrane Handbook for Systematic Reviews of Interventions [20, 21]. The protocol was registered prospectively with PROSPERO (CRD420261342862). The review question, eligibility criteria, outcome measures, and statistical approach were defined a priori before study screening and data extraction. Because this study synthesized data from previously published randomized controlled trials, institutional review board approval and informed consent were not required.

### 2.2 Data Sources and Search Strategy

A systematic literature search of PubMed/MEDLINE and the Cochrane Central Register of Controlled Trials (CENTRAL) was performed from database inception through March 2026. The search strategy combined controlled vocabulary terms and free‐text keywords related to atrial fibrillation, pulsed field ablation, radiofrequency ablation, cryoballoon ablation, thermal ablation, catheter ablation, and pulmonary vein isolation. The reference lists of eligible studies, relevant review articles, and prior meta‐analyses were also screened manually to identify additional reports. No restriction on publication year or language was applied. Duplicate records were removed before formal screening. The complete database‐specific search strategy is provided in Table S1.

### 2.3 Study Selection

Two investigators (EN and DB) independently screened all retrieved citations by title and abstract. Full‐text articles considered potentially eligible were then reviewed independently according to prespecified inclusion and exclusion criteria. Any disagreement at either stage was resolved through discussion and, when necessary, adjudication by a third reviewer (AAAC).

Eligible studies were required to meet all of the following criteria: Randomized controlled trial design; enrollment of adult patients with paroxysmal atrial fibrillation undergoing first‐time catheter ablation; direct comparison of pulsed field ablation (PFA) with conventional thermal ablation; and reporting of at least one prespecified efficacy, safety, or procedural outcome. Conventional thermal ablation was defined as radiofrequency ablation (RFA), cryoballoon ablation (CBA), or a prespecified pooled thermal control arm composed of one of these two modalities according to the original trial design. Studies were excluded if they were observational, nonrandomized, single‐arm, review articles, editorials, case reports, letters without original data, conference abstracts without sufficient extractable information, duplicate publications, or studies involving redo ablation, surgical ablation, or mixed atrial fibrillation populations without separately extractable data for paroxysmal atrial fibrillation (Table S2).

### 2.4 Quality Assessment and Data Extraction

Data extraction was performed independently by EN and DB using a standardized data collection form, and disagreements were resolved by consensus with AAAC. For each included study, the following information was abstracted: First author, year of publication, study setting, trial design, number of centers, sample size, baseline patient characteristics, intervention and comparator details, rhythm‐monitoring strategy, blanking period, follow‐up duration, and analysis population. Outcome data were extracted preferentially from the intention‐to‐treat, modified intention‐to‐treat, or full analysis set population whenever available. If multiple reports from the same trial were identified, the most complete and methodologically relevant publication was used. All baseline characteristics and clinical outcomes were extracted from the original trial reports rather than from prior reviews. For each PFA arm, we additionally extracted the catheter and sheath, generator or delivery system, waveform and pulse characteristics when reported, application pattern, mapping strategy, PVI‐verification and waiting‐period protocol, and rules for supplemental or adjunctive ablation; these details are presented in Table S7. For each trial, we also extracted the scheduled follow‐up visits, rhythm‐monitoring modality and frequency, blanking‐period definition, arrhythmia‐duration threshold, recurrence‐adjudication process, and trial‐defined serious‐adverse‐event construct and ascertainment window; these details are summarized in Table S8.

The methodological quality of the included trials was assessed independently by EN and DB using the revised Cochrane Risk of Bias tool for randomized trials (RoB 2), and disagreements were resolved by AAAC [22]. Each study was evaluated across the following domains: Bias arising from the randomization process, bias due to deviations from intended interventions, bias due to missing outcome data, bias in measurement of outcomes, and bias in selection of the reported result. Each outcome was then categorized as low risk of bias, some concerns, or high risk of bias.

### 2.5 Outcome Measures

The outcome domains and direction of effect were prespecified before study screening and data extraction. Because the original trials used nonidentical endpoint definitions, reporting windows, and ascertainment strategies, the detailed cross‐trial mapping algorithm was finalized during data extraction and before quantitative pooling. Two reviewers independently applied the mapping rules, with disagreements resolved by consensus and third‐reviewer adjudication when necessary. We preferentially used the trial‐defined primary or prespecified outcome, the intention‐to‐treat, modified intention‐to‐treat, or full‐analysis‐set population, and follow‐up through approximately 12 months. We retained each trial's original endpoint components, blanking period, and reported missing‐data handling; we did not selectively add or remove composite components, redefine events, or re‐impute participant‐level outcomes. The complete harmonization framework is presented in Table S5A.

The primary efficacy outcome was treatment success through approximately 12 months after the post‐ablation blanking period. ADVENT, BEAT PAROX‐AF, and InsightPFA contributed their trial‐defined binary primary efficacy‐success endpoints. SINGLE SHOT CHAMPION reported recurrence as its primary endpoint; therefore, freedom from primary‐endpoint recurrence between days 91 and 365 was calculated as the complement of the directly reported recurrence count to maintain a consistent favorable‐event direction. These endpoints were considered clinically aligned for comparative synthesis but were not assumed to be definitionally identical. Trial‐specific primary efficacy definitions, blanking periods, rhythm‐monitoring schedules, recurrence ascertainment, analysis populations, exact data entered, and selection rationales are detailed in Table S5B.

The secondary efficacy outcomes were repeat catheter ablation, antiarrhythmic‐drug resumption, electrical cardioversion, and recurrent atrial arrhythmias. These outcomes were pooled only when comparative binary data were directly reported or could be derived without redefining the underlying event. Trial‐specific blanking periods and reporting windows were retained. Recurrent atrial arrhythmia was mapped to the trial‐defined post‐blanking AF, AFL, or atrial tachycardia event; repeat ablation, antiarrhythmic‐drug resumption, and cardioversion were mapped to the corresponding trial‐reported patient‐level events. No composite total was reconstructed from overlapping component events. The exact trial‐level definitions and event counts used for every efficacy analysis are provided in Table S5C.

The primary safety outcome was the trial‐defined composite of serious device‐ or procedure‐related adverse events or serious procedural complications. A composite was pooled only when its clinical construct and event window were sufficiently comparable with those of the other included trials. Secondary safety outcomes were all‐cause death, stroke or transient ischemic attack, cardiac tamponade or perforation, and pulmonary vein stenosis. Individual safety outcomes were retained according to the original trial definitions and ascertainment methods; studies with zero events in both arms were documented but did not contribute to relative‐risk estimation. Procedural outcomes were total procedure time, left atrial (LA) dwell time, fluoroscopy time, cavotricuspid isthmus ablation, and general anesthesia (GA) use. GA use was treated as a procedural‐context/process measure rather than a patient‐centered efficacy or safety outcome. Continuous procedural outcomes were pooled in minutes using trial‐reported means and standard deviations, without retrospective redefinition of trial‐specific start and stop points. Binary procedural outcomes were pooled as the reported number of participants with the event. Trial‐level safety and procedural definitions, eligibility decisions, and exact data entered are provided in Table S5D. Trial‐specific follow‐up schedules, rhythm‐monitoring methods, blanking periods, recurrence definitions, and serious‐adverse‐event constructs and ascertainment windows are summarized together in Table S8.

### 2.6 Certainty of Evidence Assessment

The certainty of evidence for the principal efficacy, safety, and procedural outcomes was assessed independently by EN and DB using the Grading of Recommendations Assessment, Development and Evaluation (GRADE) framework, with disagreement resolved by AAAC [23, 24, 25]. The certainty of evidence was rated as high, moderate, low, or very low after consideration of risk of bias, inconsistency, indirectness, imprecision, and publication bias. A Summary of Findings table was prepared for the main outcomes to present the certainty rating together with the pooled effect estimates.

### 2.7 Statistical Analysis

All pooled analyses were performed using data reported according to the intention‐to‐treat principle whenever available. Dichotomous outcomes were summarized as risk ratios (RRs) with 95% confidence intervals (CIs) using the Mantel–Haenszel method. Continuous outcomes were summarized as mean differences with 95% CIs using the inverse‐variance method. Because clinical and methodological heterogeneity was anticipated across trials, including differences in PFA platform, comparator modality, rhythm‐monitoring strategy, and endpoint definition, a random‐effects model was used for the primary analyses [21]. Accordingly, pooled estimates were interpreted as average class‐level effects across the included randomized comparisons rather than as evidence that individual PFA platforms or thermal modalities were interchangeable. Trial‐level differences in catheter design, pulse characteristics, mapping, anesthesia, PVI verification, and comparator workflow are summarized in Table S6.

Statistical heterogeneity was assessed using Cochran's Q test and quantified with the *I* ^2^ statistic [21]. A 2‐sided *p* value of < 0.05 was considered statistically significant. For safety outcomes, statistical non‐significance was not interpreted as equivalence; conclusions considered the number of observed events, the width and clinically plausible range of the 95% CI, and the GRADE assessment of imprecision. For dichotomous outcomes with zero events in both treatment arms, the corresponding study was excluded from pooled relative risk estimation for that specific outcome because it did not contribute information regarding comparative effect size.

Given the limited number of eligible randomized trials, subgroup analyses and meta‐regression were not performed. Instead, leave‐one‐out sensitivity analyses were prespecified and conducted by excluding one study at a time to evaluate the robustness of the pooled estimates, particularly for outcomes with notable heterogeneity or outcomes potentially influenced by a single trial. Formal assessment of publication bias was not performed because the small number of studies per outcome would make funnel plot interpretation and related statistical testing unreliable [21]. Statistical analyses were performed using Review Manager (RevMan), version 5.4 and R version 4.5.3.

Comparator‐specific subgroup analyses were not performed because the evidence comprised only two RFA‐only trials, one cryoballoon‐only trial, and one mixed‐comparator trial (ADVENT); modality‐specific estimates would therefore have been based on too few independent trials for reliable inference [10, 11, 12, 13, 21]. Accordingly, pooled procedural outcomes were interpreted as exploratory summaries limited by comparator heterogeneity rather than as direct energy‐specific effects.

Platform‐specific subgroup analysis was not performed because three trials evaluated FARAWAVE/FARAPULSE pentaspline systems, whereas only InsightPFA evaluated the LotosPFA/InRythm nanosecond platform. A platform stratum represented by a single trial could not support reliable between‐platform inference and would remain confounded by comparator modality, mapping strategy, waiting‐period protocol, anesthesia practice, and procedural workflow [10, 11, 12, 13, 21]. Platform characteristics were therefore summarized descriptively in Table S7 rather than analyzed quantitatively.

## 3 Results

### 3.1 Study Selection

**FIGURE 1:**
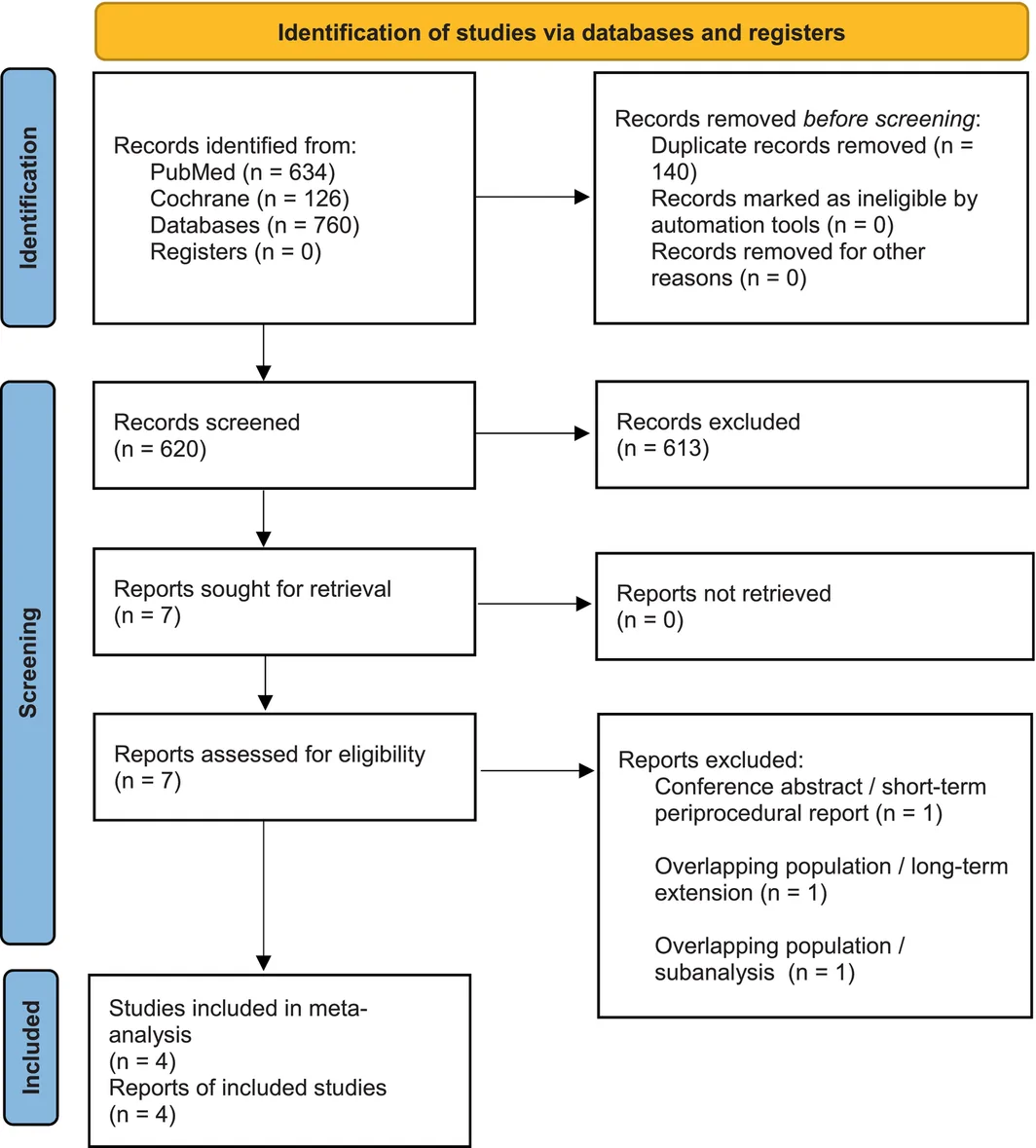
PRISMA flow diagram showing study identification, screening, full‐text eligibility assessment, and inclusion in the quantitative synthesis. PRISMA = Preferred Reporting Items for Systematic Reviews and Meta‐Analyses; RCT = randomized controlled trial.

The study selection process is summarized in Figure 1. A total of 760 records were identified through database searching, including 634 from PubMed/MEDLINE and 126 from the Cochrane Central Register of Controlled Trials. After removal of 140 duplicates, 620 records underwent title and abstract screening, of which 613 were excluded. Seven reports were assessed in full text for eligibility, and 4 randomized trials met the inclusion criteria for quantitative synthesis (Table S3). Overall, 1393 patients were included, with 697 in the PFA arm and 696 in the thermal ablation arm.

### 3.2 Baseline Characteristics

Baseline characteristics of the included trials are summarized in Table 1. All 4 trials enrolled patients with paroxysmal atrial fibrillation (AF) undergoing first catheter ablation and reported 12‐month follow‐up. ADVENT 2023 was a multicenter randomized single‐blind non‐inferiority trial comparing PFA with a conventional thermal control arm composed of radiofrequency ablation (RFA) or cryoballoon ablation (CBA) according to site assignment. SINGLE SHOT CHAMPION 2025 was a Swiss 2‐center randomized non‐inferiority trial comparing PFA with CBA. BEAT PAROX‐AF 2025 was a 9‐center European randomized superiority trial comparing PFA with point‐by‐point RFA using the CLOSE protocol. InsightPFA 2025 was a 13‐center Chinese randomized non‐inferiority trial comparing nanosecond PFA with ablation index‐guided RFA.

**TABLE 1:** Baseline characteristics of randomized trials comparing pulsed field ablation with thermal ablation.

Study	Year	Setting/design	AF type	Experimental strategy	Control strategy	*Sample* *size*, *n*	Age	Male sex	BMI, kg/m^2^	CHA_2_DS_2_‐ VASc	HTN	DM	Prior stroke/TIA/SE	AF duration	Baseline AAD use	LVEF/LA size
ADVENT	2023	Multicenter randomized, single‐blind, noninferiority	Paroxysmal AF	PFA	Thermal (RF or CBA)	305/302	62.4 ± 8.7/62.5 ± 8.5 y	66.2%/64.6%	28.3 ± 4.6/29.0 ± 4.8	1.7 ± 1.2/1.7 ± 1.2	57.0%/52.6%	10.8%/10.6%	3.9%/5.0%	3.8 ± 6.2/3.3 ± 4.5 y	98.7%/99.3%	LVEF NR; LA size NR
SINGLE SHOT CHAMPION	2025	Switzerland, 2‐center, randomized, noninferiority	Paroxysmal AF	Single‐shot PFA	Cryoballoon	105/105	64.0 ± 9.4/63.3 ± 9.6 y	73%/70%	27.0 ± 3.9/27.3 ± 4.7	2.0 (1.0–3.0)/2.0 (1.0–3.0)	53%/55%	12%/9%	4%/7%	2.4 ± 3.7/3.0 ± 4.2 y	21%/23%	LVEF 60 ± 7/60 ± 6%; LA 39 ± 5/38 ± 6 mm
BEAT PAROX‐ AF	2025	Europe, 9‐center, randomized, superiority	Paroxysmal AF	PFA	RF (CLOSE)	145/144	65.0 (56.4–70.4)/62.5 (55.1–67.2) y	57.2%/59.0%	27.2 ± 3.8/27.5 ± 4.2	1.8 ± 1.2/1.7 ± 1.2	51.7%/52.1%	10.3%/9.7%	3.4%/6.3%	12.5 (6.4–50.7)/12.6 (5.6–47.2) mo	65.5%/59.0%	LVEF 61.0 ± 5.8/61.3 ± 6.3%; LA 40.9 ± 6.7/40.3 ± 6.4 mm
InsightPFA	2025	China, 13‐center, randomized, noninferiority	Paroxysmal AF	Single‐shot PFA	AI‐guided RF	142/145	59.50 (51.00–66.00)/61.00 (54.00–67.00) y	66.90%/57.93%	24.50 (22.85–26.24)/24.61 (22.62–27.08)	1.00 (0.00–2.00)/1.00 (1.00–2.00)	40.85%/47.59%	11.27%/6.90%	2.82%/2.76%	0.82 ± 2.40/0.96 ± 3.90 y	64.08%/64.83%	LVEF 64.82 ± 5.60/65.05 ± 6.08%; LA 36.62 ± 4.75/37.01 ± 5.11 mm

*Note:* Sample sizes are shown as PFA/thermal ablation and reflect the baseline or main analysis population used for each trial in this review. Outcome‐specific denominators may differ because not all trials reported every outcome, and some trials used endpoint‐specific analysis populations. Where a trial reported medians or IQRs in its baseline table, those values were preserved rather than converted. ADVENT main baseline table did not report LVEF or left atrial size, so those fields are marked NR rather than imputed.

Abbreviations: AAD, antiarrhythmic drug; AF, atrial fibrillation; AI, ablation index; CBA, cryoballoon; HTN, hypertension; LA, left atrial; LVEF, left ventricular ejection fraction; NR, not reported; PFA, pulsed field ablation; RF, radiofrequency; SE, systemic embolism; TIA, transient ischemic attack.

Detailed PFA platform characteristics are presented in Table S7. ADVENT, SINGLE SHOT CHAMPION, and BEAT PAROX‐AF evaluated FARAWAVE/FARAPULSE pentaspline systems, whereas InsightPFA evaluated the distinct LotosPFA/InRythm nanosecond platform. Catheter configuration, pulse delivery, mapping, PVI verification, waiting periods, and adjunctive‐ablation protocols were not uniform across trials [10, 11, 12, 13].

Across the 4 trials, baseline characteristics were generally balanced between treatment arms within individual trials. Mean or median age ranged from 59.5 to 65.0 years in the PFA arms and from 61.0 to 63.3 years in the thermal ablation arms. Men comprised 57.9% to 73.0% of participants. Baseline body mass index (BMI) ranged from 24.5 to 29.0 kg/m^2^, and baseline CHA_2_DS_2_‐VASc burden was low across trials. Hypertension was present in 40.9% to 57.0%, diabetes mellitus in 6.9% to 11.3%, and prior stroke, transient ischemic attack, or systemic embolism in 2.8% to 7.0%. Baseline antiarrhythmic drug use ranged from 21.0% to 99.3%. In the trials that reported echocardiographic parameters, left ventricular ejection fraction (LVEF) was preserved, ranging from 60.0% to 65.1%, and LA size ranged from 36.6 to 40.9 mm. ADVENT did not report baseline LVEF or LA size in the main baseline table.

### 3.3 Efficacy Outcomes

#### 3.3.1 12‐Month Treatment Success

**FIGURE 2:**
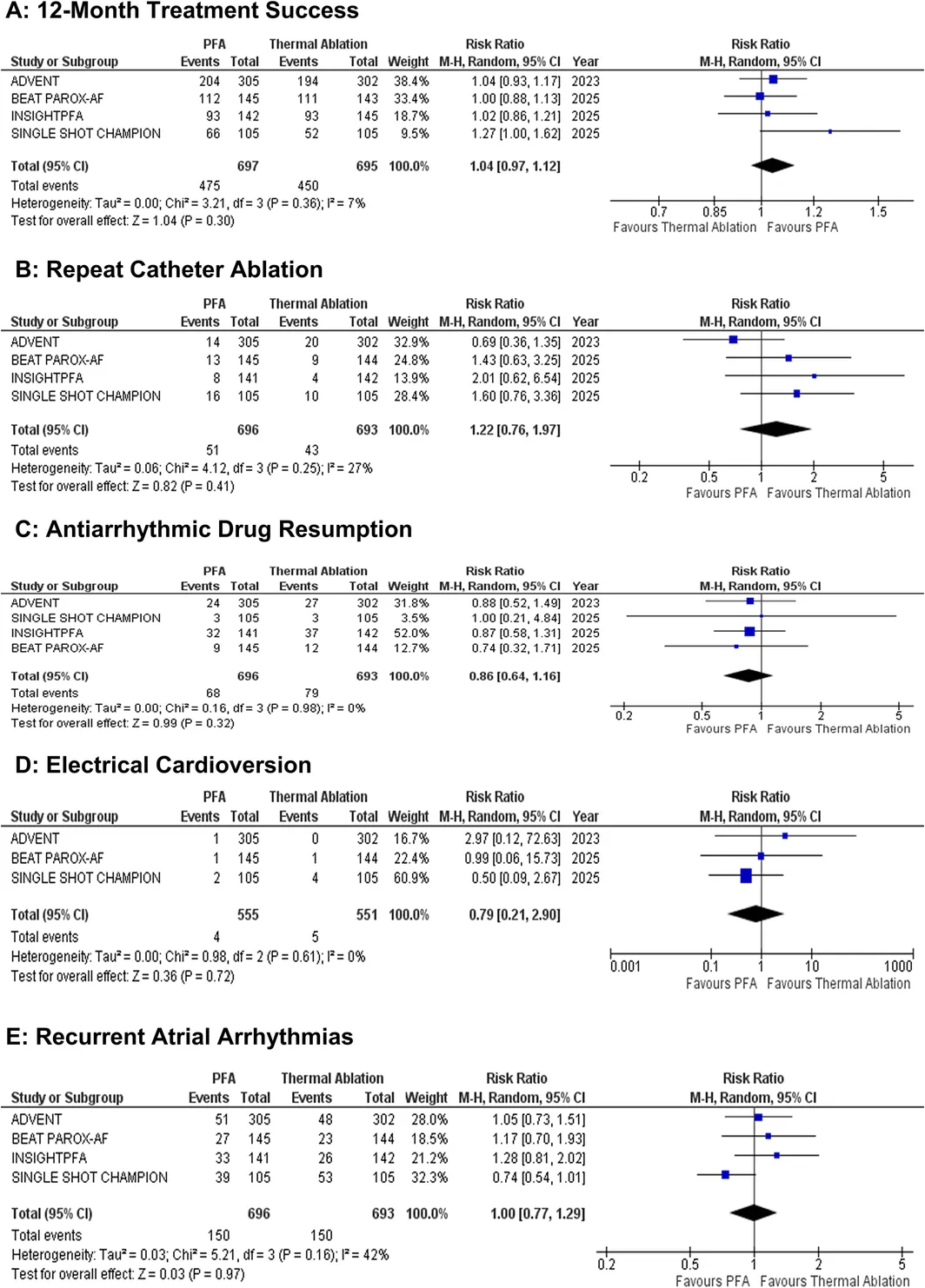
Forest plots for efficacy outcomes. Forest plots comparing pulsed field ablation with thermal ablation for (A) 12‐month treatment success, (B) repeat catheter ablation, (C) antiarrhythmic drug resumption after the blanking period, (D) electrical cardioversion after the blanking period, and (E) recurrent atrial arrhythmias. AAD = antiarrhythmic drug; CI = confidence interval; PFA = pulsed field ablation; RR = risk ratio.

Four trials comprising 1 392 patients reported 12‐month treatment success. A total of 475 events occurred among 697 patients in the PFA arm and 450 events among 695 patients in the thermal ablation arm. Based on the pooled estimate, there was no statistically significant difference between PFA and thermal ablation for 12‐month treatment success (RR, 1.04; 95% CI, 0.97 to 1.12; *p* = 0.30; *I* ^2^ = 7%; Figure 2A). Although statistical heterogeneity was low, this analysis combined clinically aligned but nonidentical trial‐specific efficacy constructs, with different blanking periods and rhythm‐monitoring intensities (Table S5B and Table S5C). Because SINGLE SHOT CHAMPION used continuous implantable cardiac monitoring whereas the other trials used intermittent surveillance, this pooled estimate should be interpreted as a relative effect across nonuniform ascertainment strategies rather than as a common absolute success rate [10, 11, 12, 13] (Table S8).

#### 3.3.2 Repeat Catheter Ablation

Four trials comprising 1 389 patients reported repeat catheter ablation. A total of 51 events occurred among 696 patients in the PFA arm vs. 43 events among 693 patients in the thermal ablation arm. The pooled effect was not statistically significant between the two groups (RR, 1.22; 95% CI, 0.76 to 1.97; *p* = 0.41; *I* ^2^ = 27%; Figure 2B).

#### 3.3.3 Antiarrhythmic Drug Resumption

Four trials comprising 1 389 patients reported antiarrhythmic drug resumption after the blanking period. A total of 68 events occurred among 696 patients in the PFA arm vs. 79 events among 693 patients in the thermal ablation arm. There was no statistically significant difference between the two groups (RR, 0.86; 95% CI, 0.64 to 1.16; *p* = 0.32; *I* ^2^ = 0%; Figure 2C).

#### 3.3.4 Electrical Cardioversion

Three trials comprising 1 106 patients reported electrical cardioversion. A total of 4 events occurred among 555 patients in the PFA arm vs. 5 events among 551 patients in the thermal ablation arm. The pooled effect was not statistically significant between the two groups (RR, 0.79; 95% CI, 0.21 to 2.90; *p* = 0.72; *I* ^2^ = 0%; Figure 2D).

#### 3.3.5 Recurrent Atrial Arrhythmias

Four trials comprising 1 389 patients reported recurrent atrial arrhythmias. A total of 150 events occurred among 696 patients in the PFA arm vs. 150 events among 693 patients in the thermal ablation arm. There was no statistically significant difference between the two groups (RR, 1.00; 95% CI, 0.77 to 1.29; *p* = 0.97; *I* ^2^ = 42%; Figure 2E). Monitoring intensity was not uniform: SINGLE SHOT CHAMPION used continuous implantable monitoring, whereas the other trials used scheduled and symptom‐triggered intermittent recordings. Accordingly, the pooled recurrence estimate should be interpreted cautiously because asymptomatic episodes were more likely to be detected under continuous surveillance [10, 11, 12, 13] (Table S8).

### 3.4 Safety Outcomes

#### 3.4.1 Composite Serious Adverse Events

**FIGURE 3:**
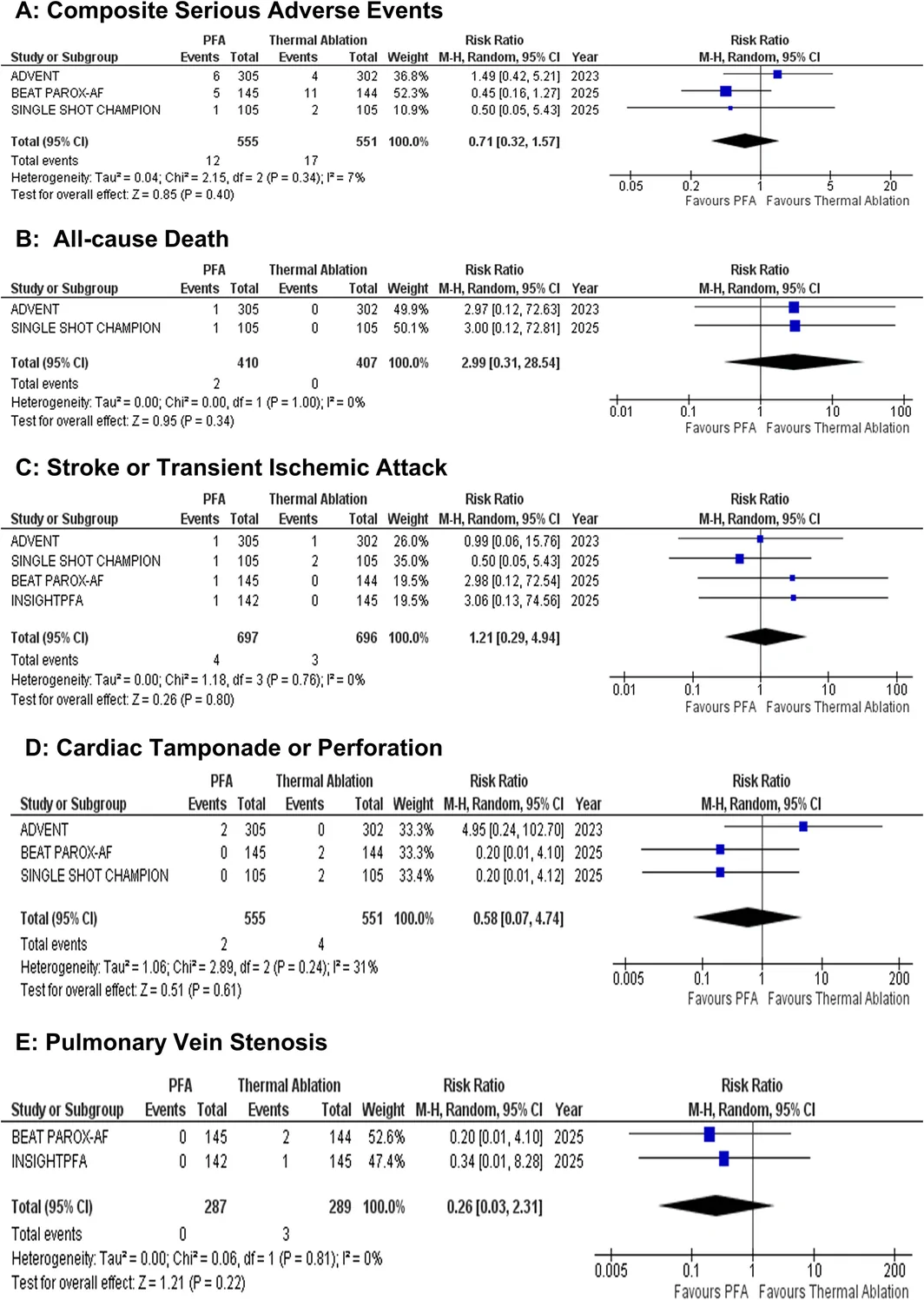
Forest plots for safety outcomes. Forest plots comparing pulsed field ablation with thermal ablation for (A) composite serious adverse events, (B) all‐cause death, (C) stroke or transient ischemic attack, (D) cardiac tamponade or perforation, and (E) pulmonary vein stenosis. CI = confidence interval; PFA = pulsed field ablation; RR = risk ratio; TIA = transient ischemic attack.

Three trials comprising 1 106 patients reported composite serious adverse events. A total of 12 events occurred among 555 patients in the PFA arm and 17 events among 551 patients in the thermal ablation arm. Based on the pooled estimate across the 3 trials, there was no statistically significant difference between the two groups (RR, 0.71; 95% CI, 0.32 to 1.57; *p* = 0.40; *I* ^2^ = 7%; Figure 3A), but the wide CI was compatible with both a substantial reduction and a clinically important increase in serious adverse events; therefore, equivalence cannot be inferred. In addition, the contributing trials used nonidentical serious‐adverse‐event definitions and ascertainment windows; the pooled composite therefore represents clinically related, but not definitionally uniform, safety constructs [10, 11, 12] (Table S8).

#### 3.4.2 All‐Cause Death

Two trials comprising 817 patients reported all‐cause death. A total of 2 events occurred among 410 patients in the PFA arm, whereas no events occurred among 407 patients in the thermal ablation arm. Because only 2 deaths were observed, the comparative estimate was extremely imprecise (RR, 2.99; 95% CI, 0.31 to 28.54; *p* = 0.34; *I* ^2^ = 0%; Figure 3B), and no reliable conclusion regarding a difference or equivalence between strategies can be drawn.

#### 3.4.3 Stroke or Transient Ischemic Attack

Four trials comprising 1 393 patients reported stroke or transient ischemic attack. A total of 4 events occurred among 697 patients in the PFA arm vs. 3 events among 696 patients in the thermal ablation arm. No statistically detectable difference was observed (RR, 1.21; 95% CI, 0.29 to 4.94; *p* = 0.80; *I* ^2^ = 0%; Figure 3C); however, only 7 total events occurred, and the wide confidence interval did not exclude clinically important benefit or harm.

#### 3.4.4 Cardiac Tamponade or Perforation

Three trials comprising 1 106 patients reported cardiac tamponade or perforation. A total of 2 events occurred among 555 patients in the PFA arm vs. 4 events among 551 patients in the thermal ablation arm. No statistically detectable difference was observed (RR, 0.58; 95% CI, 0.07 to 4.74; *p* = 0.61; *I* ^2^ = 31%; Figure 3D); however, the estimate was based on only 6 total events and was too imprecise to establish equivalence or exclude a clinically important difference.

#### 3.4.5 Pulmonary Vein Stenosis

Two trials comprising 576 patients reported pulmonary vein stenosis. No events occurred among 287 patients in the PFA arm, whereas 3 events occurred among 289 patients in the thermal ablation arm. No statistically detectable difference was observed (RR, 0.26; 95% CI, 0.03 to 2.31; *p* = 0.22; *I* ^2^ = 0%; Figure 3E); however, the estimate was based on only 3 events and remained too imprecise to support a definitive comparative or equivalence conclusion.

### 3.5 Procedural Outcomes

#### 3.5.1 Total Procedure Time

**FIGURE 4:**
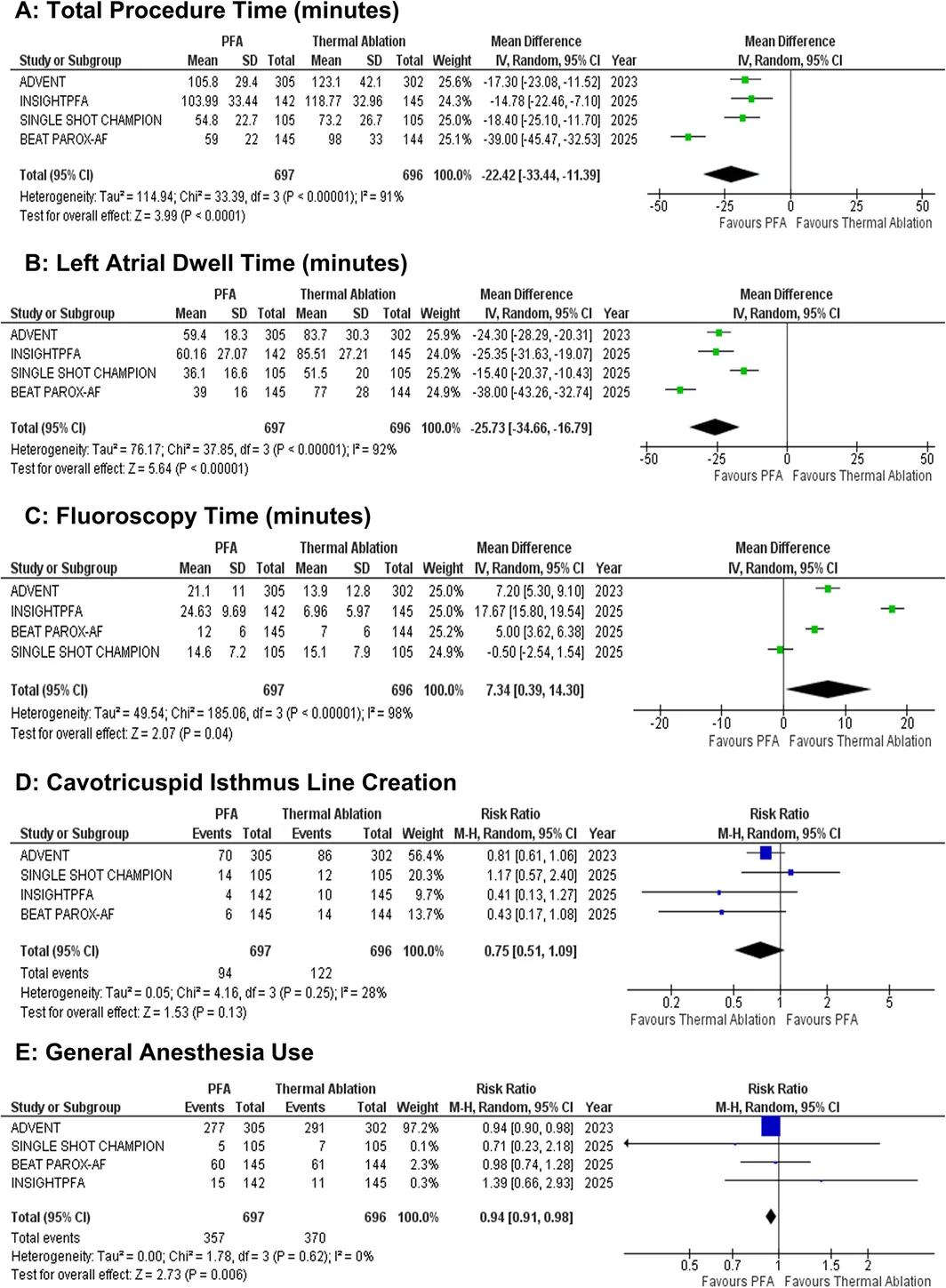
Forest plots for procedural outcomes. Forest plots comparing pulsed field ablation with thermal ablation for (A) total procedure time, (B) left atrial dwell time, (C) fluoroscopy time, (D) cavotricuspid isthmus line creation, and (E) general anesthesia use. CI = confidence interval; CTI = cavotricuspid isthmus; GA = general anesthesia; LA = left atrial; MD = mean difference; PFA = pulsed field ablation; RR = risk ratio.

Four trials comprising 1 393 patients reported total procedure time. Based on the pooled estimate across the 4 trials, PFA was associated with a significantly shorter total procedure time compared with thermal ablation (MD, −22.42 min; 95% CI, −33.44 to −11.39; *p* < 0.0001; *I* ^2^ = 91%; Figure 4A).

#### 3.5.2 LA Dwell Time

Four trials comprising 1 393 patients reported LA dwell time. The pooled estimate showed a significantly shorter LA dwell time with PFA compared with thermal ablation (MD, −25.73 min; 95% CI, −34.66 to −16.79; *p* < 0.00001; *I* ^2^ = 92%; Figure 4B).

#### 3.5.3 Fluoroscopy Time

Four trials comprising 1 393 patients reported fluoroscopy time. PFA was associated with significantly longer fluoroscopy time compared with thermal ablation (MD, 7.34 min; 95% CI, 0.39 to 14.30; *p* = 0.04; *I* ^2^ = 98%; Figure 4C).

#### 3.5.4 Cavotricuspid Isthmus Line Creation

Four trials comprising 1 393 patients reported cavotricuspid isthmus line creation. A total of 94 events occurred among 697 patients in the PFA arm and 122 events among 696 patients in the thermal ablation arm. Based on the pooled estimate, there was no statistically significant difference between PFA and thermal ablation for cavotricuspid isthmus line creation (RR, 0.75; 95% CI, 0.51 to 1.09; *p* = 0.13; *I* ^2^ = 28%; Figure 4D).

#### 3.5.5 General Anesthesia Use

Four trials comprising 1 393 patients reported GA use. GA was used in 357 of 697 patients (51.2%) in the PFA arm and 370 of 696 patients (53.2%) in the thermal ablation arm, corresponding to a crude absolute difference of 1.9 percentage points lower with PFA. The pooled estimate was statistically significant (RR, 0.94; 95% CI, 0.91 to 0.98; *p* = 0.006; *I* ^2^ = 0%; Figure 4E). However, because anesthesia selection was not standardized and varied with trial protocol, institutional practice, operator preference, and procedural workflow, this finding was interpreted as a practice‐pattern signal rather than a clinically meaningful or energy‐specific advantage of PFA (Table S5D and Table S6).

Comparator‐specific subgroup analyses were not undertaken for procedural outcomes because the available evidence comprised two RFA‐only comparisons, one cryoballoon‐only comparison, and one mixed‐comparator trial. Consequently, the pooled procedure‐time, LA dwell‐time, fluoroscopy‐time, CTI‐ablation, and GA‐use estimates were treated as exploratory and limited by comparator heterogeneity, not as direct energy‐specific effects [10, 11, 12, 13] (Table S6).

### 3.6 Leave‐One‐Out Analysis

For total procedure time, heterogeneity was eliminated after omission of BEAT PAROX‐AF (*I* ^2^ = 0.0%), whereas heterogeneity remained high after omission of ADVENT, InsightPFA, and SINGLE SHOT CHAMPION (*I* ^2^ = 93.0%, 93.0%, and 93.6%, respectively). This suggests that BEAT PAROX‐AF was the main contributor to between‐study heterogeneity for total procedure time, while the pooled effect remained consistently in favor of PFA across all leave‐one‐out analyses (Figure S1).

For fluoroscopy time, heterogeneity remained substantial across all leave‐one‐out analyses, with *I* ^2^ values ranging from 93.6% to 98.9%. The greatest reduction was observed after omission of InsightPFA (*I* ^2^ = 93.6%), although heterogeneity remained substantial, suggesting that between‐study variability was not explained by a single study alone (Figure S2).

For LA dwell time, heterogeneity remained high across all leave‐one‐out analyses, with I^2^ values ranging from 78.0% to 94.7%. The greatest reduction was observed after omission of BEAT PAROX‐AF (I^2^ = 78.0%), suggesting partial contribution from this study. However, the persistence of high heterogeneity after all study omissions indicates that variability was unlikely to be explained by a single study alone (Figure S3).

### 3.7 Risk of Bias

Risk of bias is summarized in Figure S4. ADVENT 2023 was judged as having some concerns overall, driven by some concerns in bias arising from the randomization process and bias due to missing outcome data. SINGLE SHOT CHAMPION 2025 also had some concerns in the randomization domain. In contrast, BEAT PAROX‐AF 2025 and InsightPFA 2025 were judged as having low risk of bias across all assessed domains. Bias due to deviations from intended interventions, bias in measurement of the outcome, and bias in selection of the reported result were judged as low risk in all or nearly all trials.

### 3.8 Certainty of Evidence

The certainty of evidence for the prespecified outcomes is summarized in Table S4. Certainty was moderate for repeat catheter ablation and for the observed association in GA use; however, GA use was treated as a contextual process measure rather than a patient‐centered benefit. Certainty was low for 12‐month treatment success, antiarrhythmic drug resumption, recurrent atrial arrhythmias, composite serious adverse events, pulmonary vein stenosis, and cavotricuspid isthmus line creation. Certainty was very low for electrical cardioversion, all‐cause death, stroke or transient ischemic attack, cardiac tamponade or perforation, total procedure time, LA dwell time, and fluoroscopy time.

Across outcomes, downgrading was driven primarily by risk of bias, imprecision, and inconsistency. Imprecision was the main reason for downgrading rare clinical safety outcomes, whereas substantial heterogeneity contributed importantly to downgrading of total procedure time, LA dwell time, and fluoroscopy time. Accordingly, nonsignificant safety estimates were interpreted as inconclusive rather than as evidence of equivalence.

## 4 Discussion

We report several key findings in this meta‐analysis. Across four randomized trials, the evaluated pulsed field ablation (PFA) strategies showed broadly similar 12‐month relative efficacy compared with their trial‐specific thermal comparators in patients undergoing first catheter ablation for paroxysmal atrial fibrillation (AF). No statistically detectable differences were observed in recurrent atrial arrhythmias, repeat catheter ablation, antiarrhythmic drug (AAD) resumption, or electrical cardioversion. Safety events were sparse, and the corresponding estimates remained imprecise. Pooled procedure and left atrial (LA) dwell times were shorter, whereas fluoroscopy time was longer with PFA. However, these procedural estimates were derived from trials using different PFA platforms, thermal comparators, and procedural workflows. They should therefore be interpreted as average effects across the included trial configurations rather than as evidence that every PFA system provides the same procedural advantage over radiofrequency or cryoballoon ablation [10, 11, 12, 13].

The efficacy findings are consistent with the individual randomized trials included in this analysis. In ADVENT, PFA was noninferior to conventional thermal ablation for 1‐year treatment success and device‐related or procedure‐related serious adverse events [10]. In SINGLE SHOT CHAMPION, PFA was noninferior to cryoballoon ablation (CBA) for atrial tachyarrhythmia recurrence when recurrence was assessed with continuous rhythm monitoring [11]. In BEAT PAROX‐AF, PFA did not show superior 12‐month single‐procedure success compared with point‐by‐point radiofrequency ablation (RFA) using the CLOSE protocol [12]. In InsightPFA, nanosecond PFA was noninferior to ablation index‐guided RFA for 12‐month treatment success, with no statistically detectable difference in the trial‐defined safety outcomes [13]. Therefore, the present pooled results support broadly similar relative 12‐month efficacy rather than superior efficacy of PFA over thermal ablation. Nevertheless, absolute treatment‐success and recurrence rates should not be compared uncritically across trials because endpoint composition and rhythm‐surveillance intensity differed. In particular, SINGLE SHOT CHAMPION used continuous implantable cardiac monitoring, which was more sensitive for detecting asymptomatic atrial tachyarrhythmia recurrence than the intermittent monitoring schedules used in the other trials. Consequently, the pooled result should be interpreted as a comparative treatment‐effect estimate across clinically aligned trial‐specific endpoints rather than as a uniform absolute success rate. Similar cautions apply to the secondary efficacy components because the original trials used different blanking periods and reporting windows for recurrence, antiarrhythmic drug resumption, cardioversion, and repeat ablation. We therefore retained the randomized within‐trial comparisons while explicitly documenting, rather than erasing, these design differences in Table S5B and Table S5C. Low statistical heterogeneity does not eliminate this ascertainment heterogeneity; both the pooled 12‐month treatment‐success and recurrent‐arrhythmia estimates therefore require cautious interpretation [10, 11, 12, 13] (Table S8).

These findings should be interpreted in the context of prior syntheses. Amin et al. reported lower AF recurrence and shorter total procedure time with PFA, together with lower pooled risks of phrenic nerve palsy and esophageal lesions but a higher pooled incidence of pericardial tamponade. However, their analysis combined one RCT with 16 nonrandomized studies, included both paroxysmal and persistent AF, and pooled RFA and CBA comparators [16]. Esteves et al. reported comparable 1‐year success and major complication rates, shorter procedure time, and longer fluoroscopy time with PFA vs. RFA in paroxysmal AF, but their analysis included one RCT and five observational studies [17]. The randomized‐only analysis by Krishan et al. was limited to ADVENT and SINGLE SHOT CHAMPION, whereas Mojica et al. evaluated a broader randomized evidence base that included persistent AF, a dual‐energy PFA and RFA trial, and a 24‐h physiology‐focused trial [18, 19]. The incremental contribution of the present study lies in its more focused clinical and methodological framework. Eligibility was restricted to dedicated RCTs of first‐ablation paroxysmal AF, including the newer BEAT PAROX‐AF and InsightPFA trials, and outcome interpretation was supported by trial‐level mapping of endpoint definitions, rhythm‐monitoring strategies, safety ascertainment, platform characteristics, comparator modalities, anesthesia practices, and procedural workflows. This framework provides a clinically focused and transparently characterized synthesis of the current randomized evidence, distinguishing broadly similar short‐term efficacy estimates from safety findings limited by sparse events and imprecision and from procedural findings influenced by substantial platform, comparator, and workflow heterogeneity [10, 11, 12, 13, 14, 15, 16, 17, 18, 19]. A structured comparison of relevant prior syntheses is provided in Table S9.

Clinical comparability should be understood as a class‐level summary of randomized within‐trial contrasts, not as proof of technological interchangeability. Radiofrequency ablation and cryoballoon ablation are established but procedurally distinct thermal approaches; although FIRE AND ICE demonstrated noninferior clinical efficacy of cryoballoon ablation relative to RFA, the two technologies differ in energy delivery, catheter manipulation, mapping requirements, and procedural workflow [5]. These distinctions are relevant to the present synthesis. ADVENT compared a microsecond pentaspline PFA system with a site‐specific mixed thermal control comprising either contact‐force RFA or cryoballoon ablation; SINGLE SHOT CHAMPION compared pentaspline PFA directly with cryoballoon ablation; BEAT PAROX‐AF compared pentaspline PFA with optimized point‐by‐point RFA using the CLOSE protocol and electroanatomic mapping; and InsightPFA compared a distinct nanosecond biphasic LotosPFA system with ablation index‐guided RFA [10, 11, 12, 13] (Table S6). Low statistical heterogeneity for treatment success does not remove these clinical and technological differences. The pooled efficacy estimate therefore indicates broadly similar relative 12‐month performance across the included trial‐specific comparisons, but it does not establish interchangeability among PFA platforms or uniform comparability with both RFA and cryoballoon ablation. Platform‐specific subgroup analysis was not feasible because InsightPFA was the only trial evaluating the LotosPFA/InRythm nanosecond system, whereas the remaining trials used FARAWAVE/FARAPULSE pentaspline platforms. Any quantitative cross‐platform contrast would therefore be driven by a single trial and remain inseparable from comparator, mapping, waiting‐period, anesthesia, and workflow differences [10, 11, 12, 13, 21] (Table S7).

The safety findings are clinically important, but they require cautious interpretation. PFA produces irreversible electroporation and has shown preferential myocardial effects in preclinical and clinical mechanistic studies [6, 7]. Esophageal sparing has also been reported after PFA‐guided PVI [6]. These data provide a mechanistic rationale for evaluating PFA as an alternative to thermal ablation. However, the present randomized evidence remains insufficiently precise for rare complications. Large observational datasets, including MANIFEST‐17K, PULSED AF, and MANIFEST‐PF, reported low rates of major complications with PFA in clinical practice [8, 9, 26]. These studies provide external safety context, but they do not replace randomized comparisons. In the present analysis, no statistically detectable differences were observed for the composite or individual safety outcomes; however, event counts were sparse, and the confidence intervals were wide. These estimates should therefore be regarded as inconclusive rather than as evidence of equivalent safety, because clinically important benefit or harm could not be excluded. Consistent with this interpretation, the GRADE certainty was low for composite serious adverse events and pulmonary vein stenosis and very low for all‐cause death, stroke or transient ischemic attack, and cardiac tamponade or perforation, principally because of imprecision. Safety interpretation is additionally limited by nonuniform endpoint construction: ADVENT and BEAT PAROX‐AF used prespecified device‐ or procedure‐related serious‐adverse‐event composites with a 7‐day window, with atrioesophageal fistula and pulmonary vein stenosis counted regardless of timing; SINGLE SHOT CHAMPION used a 30‐day composite of procedure‐related complications; and InsightPFA reported a broader trial‐period safety framework that was not entered into the pooled composite [10, 11, 12, 13] (Table S8).

Procedural efficiency is highly context‐dependent. ADVENT combined two distinct thermal comparator workflows; SINGLE SHOT CHAMPION compared two single‐shot technologies; BEAT PAROX‐AF benchmarked PFA against standardized CLOSE‐guided point‐by‐point RFA; and InsightPFA evaluated a nanosecond over‐the‐wire PFA catheter against ablation index‐guided RFA, with different mapping requirements between treatment arms [10, 11, 12, 13] (Table S6). These differences may affect catheter manipulation, mapping and PVI‐verification steps, energy‐delivery time, fluoroscopy dependence, anesthesia practice, waiting periods, adjunctive ablation, and overall laboratory workflow. Therefore, pooled procedural estimates reflect average differences across the evaluated trial configurations rather than the intrinsic performance of a uniform PFA class against a uniform thermal‐ablation class. Because this comparator structure would yield only two RFA‐only trials and one cryoballoon‐only trial, while ADVENT could not be assigned to a single comparator stratum, formal comparator‐specific subgroup analyses were not performed [10, 11, 12, 13, 21].

Accordingly, shorter pooled procedure and LA dwell times should be regarded as exploratory average workflow effects rather than intrinsic energy‐specific advantages of PFA. The very high heterogeneity for procedure time, LA dwell time, and fluoroscopy time, together with the leave‐one‐out findings, supports this interpretation. Fluoroscopy exposure may be particularly influenced by mapping integration, over‐the‐wire deployment, catheter design, operator experience, anesthesia protocols, and institutional practice [10, 11, 12, 13]. The health‐system value of shorter procedure duration also depends on device cost, staffing, mapping use, reimbursement, and other local resource considerations; an ADVENT‐based economic evaluation identified procedure‐related costs as an important determinant of comparative cost‐effectiveness [27]. Thus, the pooled procedural estimates should not be generalized across all PFA devices, RFA workflows, or cryoballoon protocols. They should be interpreted alongside the technology and resource environment in which the procedure is performed and regarded as hypothesis‐generating rather than as direct energy‐specific effects.

GA use warrants separate interpretation as a procedural‐context variable. Although the pooled RR was statistically significant, the crude absolute difference was only 1.9 percentage points (51.2% vs. 53.2%). The estimate was dominated by ADVENT, which contributed 97.2% of the meta‐analytic weight and used GA in more than 90% of both treatment arms (Figure 4E). Anesthesia practice varied substantially across the remaining trials: SINGLE SHOT CHAMPION routinely used deep sedation and reserved GA for patients at higher risk from sedation; BEAT PAROX‐AF initially used conscious sedation and introduced GA at two centers; and InsightPFA permitted investigator‐selected conscious sedation or GA, with conscious sedation used in approximately 90% of both groups [10, 11, 12, 13] (Table S6). Consequently, lower pooled GA use should be regarded as a trial‐ and practice‐dependent process signal, not a patient‐centered clinical benefit or an energy‐specific advantage of PFA.

Current guidelines support catheter ablation in appropriately selected patients with symptomatic AF and recognize PVI as the central procedural target for paroxysmal AF [2, 3]. The 2024 EHRA/HRS/APHRS/LAHRS expert consensus statement discusses emerging ablation technologies, including PFA, without establishing uniform superiority of PFA over conventional thermal approaches [28]. The present results are consistent with this cautious position. The evaluated PFA systems may be considered effective nonthermal alternatives for PVI within the clinical and procedural settings represented by the available randomized trials [10, 11, 12, 13]. Any potential procedural advantage is likely to be platform‐ and workflow‐specific; treatment selection should therefore consider the particular catheter system, comparator technology, mapping strategy, operator experience, patient anatomy, anesthesia requirements, and institutional workflow.

## 5 Limitations

This study has several limitations. First, only 4 randomized trials were included, and rare safety events were sparse, producing wide CIs that preclude definitive comparative or equivalence conclusions. Second, catheter design, pulse duration and waveform, mapping integration, anesthesia practice, PVI verification, adjunctive ablation, and thermal comparator technology differed across trials, precluding platform‐specific conclusions [10, 11, 12, 13]. Third, rhythm monitoring was not uniform, with continuous implantable monitoring in SINGLE SHOT CHAMPION and intermittent monitoring in the other trials [10, 11, 12, 13]. Fourth, endpoint definitions, blanking‐period rules, AAD use, and serious adverse‐event definitions were not identical across studies [10, 11, 12, 13]. Fifth, follow‐up was limited to 12 months, so long‐term durability, late pulmonary‐vein reconnection, repeat‐procedure findings, and late rare complications could not be assessed. Sixth, high heterogeneity for total procedure time, LA dwell time, and fluoroscopy time suggests that procedural outcomes are influenced by workflow, platform, comparator modality, mapping strategy, and operator‐ or center‐level differences. Seventh, because the comparator structure comprised two RFA‐only trials, one cryoballoon‐only trial, and one mixed‐comparator trial, reliable modality‐specific subgroup synthesis was not feasible. Eighth, platform‐specific subgroup analysis was not feasible because only one RCT evaluated the LotosPFA/InRythm nanosecond platform; accordingly, this meta‐analysis cannot compare the relative performance of individual PFA platforms or pulse‐delivery technologies [10, 11, 12, 13]. These surveillance and safety‐definition differences are detailed in Table S8 and limit interpretation of pooled absolute recurrence rates and composite safety estimates.

## 6 Conclusions

In this meta‐analysis of randomized evidence, the evaluated PFA strategies showed broadly similar 12‐month efficacy to their trial‐specific thermal comparators in patients with paroxysmal AF. Sparse safety events and wide confidence intervals preclude equivalence claims and do not exclude clinically important differences. Pooled procedure and LA dwell times favored PFA, whereas fluoroscopy time favored thermal ablation; however, these estimates average heterogeneous catheters, pulse characteristics, mapping strategies, anesthesia protocols, and RFA or cryoballoon workflows and should not be interpreted as uniform platform‐specific advantages. Larger head‐to‐head randomized studies with platform‐specific reporting, standardized procedural definitions and monitoring, longer follow‐up, and sufficient power for rare complications are needed. These pooled procedural findings should therefore be considered exploratory rather than direct energy‐specific comparisons. The efficacy estimates should also be interpreted in light of nonuniform rhythm surveillance across trials. This updated synthesis therefore refines the interpretation of the randomized evidence by distinguishing relatively consistent efficacy findings from safety estimates limited by imprecision and procedural estimates limited by substantial heterogeneity and very low certainty.

## Author Contributions

Asad Ali Ahmed Cheema: Conceptualization, Methodology, Software, Validation, Formal analysis, Writing – original draft, Writing – review and editing, Visualization, Supervision, Project administration. Reshail Asif: Conceptualization, Investigation, Data curation, Writing – original draft, Writing – review and editing. Unsa Arif: Conceptualization, Investigation, Data curation, Writing – original draft, Writing – review and editing. Manahil Malik: Investigation, Data curation, Writing – original draft, Writing – review and editing. Sarah Misbah: Investigation, Writing – original draft, Writing – review and editing. Eraj Nadeem: Methodology, Validation, Investigation, Data curation, Writing – original draft, Writing – review and editing. Muhammad Iftikhar Alam: Methodology, Software, Validation, Formal analysis, Writing – original draft, Writing – review and editing, Visualization. Deep Birjani: Methodology, Validation, Investigation, Data curation, Writing – original draft, Writing – review and editing. Syed Umair Ahmed: Validation, Formal analysis, Writing – review and editing, Visualization. FNU Hamna: Investigation, Data curation, Writing – review and editing. Hadia Haseeb Ahmad: Investigation, Data curation, Writing – original draft, Writing – review and editing. Fizza Sajid: Investigation, Data curation, Writing – original draft, Writing – review and editing. Shahrukh Nadeem: Writing – review and editing, Supervision, Project administration. Zaitoon Hanif: Investigation, Writing – review and editing. Abu Huraira Bin Gulzar: Supervision, Investigation, Writing – review and editing.

## Funding

This research received no specific grant from any funding agency in the public, commercial, or not‐for‐profit sectors.

## Disclosure

Generative AI or AI‐assisted tools were used solely to check grammar, improve language clarity, and enhance the overall flow of the manuscript. The authors carefully reviewed and verified all content, interpretations, references, and conclusions, and take full responsibility for the accuracy and integrity of the final manuscript.

## Ethics Statement

The authors have nothing to report.

## Consent

The authors have nothing to report.

## Conflicts of Interest

The authors declare no conflicts of interest.

## Supporting information


**Figure S1:** Forest plot for leave‐one‐out sensitivity analysis of total procedure time. Each row shows the pooled mean difference after omitting the named study. Values to the left favor PFA; values to the right favor thermal ablation. Abbreviations: CI = confidence interval; MD = mean difference; PFA = pulsed field ablation.
**Figure S2:** Forest plot for leave‐one‐out sensitivity analysis of fluoroscopy time. Each row shows the pooled mean difference after omitting the named study. Values to the left favor PFA; values to the right favor thermal ablation. Abbreviations: CI = confidence interval; MD = mean difference; PFA = pulsed field ablation.
**Figure S3:** Forest plot for leave‐one‐out sensitivity analysis of left atrial dwell time. Each row shows the pooled mean difference after omitting the named study. Values to the left favor PFA; values to the right favor thermal ablation. Abbreviations: CI = confidence interval; LA = left atrial; MD = mean difference; PFA = pulsed field ablation.
**Figure S4:** Risk‐of‐bias assessment for included randomized controlled trials using the revised Cochrane Risk of Bias 2 tool. Domains are shown as D1 to D5: D1, bias arising from the randomization process; D2, bias due to deviations from intended interventions; D3, bias due to missing outcome data; D4, bias in measurement of the outcome; D5, bias in selection of the reported result. Green circles indicate low risk of bias and yellow circles indicate some concerns. Abbreviations: RoB 2 = Risk of Bias 2.
**Table S1:** Detailed literature search strategies.
**Table S2:** PICO eligibility criteria.
**Table S3:** Full‐text reviewed studies.
**Table S4:** GRADE evidence profile for pulsed field ablation vs. thermal ablation in paroxysmal atrial fibrillation.
**Table S5:** (A) Outcome harmonization framework applied before quantitative pooling. (B) Trial‐specific efficacy definitions, rhythm monitoring, and mapping to the pooled primary treatment‐success outcome. (C) Trial‐level mapping and exact data entered for all pooled efficacy outcomes. (D) Trial‐level mapping and exact data entered for all pooled safety and procedural outcomes.
**Table S6:** Trial‐level PFA platforms, comparator technologies, and procedural workflows.
**Table S7:** Trial‐specific PFA platforms, pulse‐delivery characteristics, mapping strategies, and adjunctive ablation protocols.
**Table S8:** Trial‐specific rhythm monitoring, follow‐up, recurrence ascertainment, and serious adverse‐event definitions.
**Table S9:** Incremental clinical and methodological contribution relative to prior PFA meta‐analyses.

## Data Availability

All data analyzed in this systematic review and meta‐analysis were extracted from the published trial reports and their associated Supporting Information.
