# Supplementary material for: Clinical Outcomes of Pulsed Field Vs. Thermal Ablation for Paroxysmal Atrial Fibrillation: Systematic Review and Meta‐Analysis of Randomized Evidence
Source: J Arrhythm. 2026 Aug 20;42(4):e70446. doi: 10.1002/joa3.70446 (PMC13491151; doi:10.1002/joa3.70446)
Supplement: Supplementary file 1 [file JOA3-42-e70446-s001.docx]

**Supplemental Material**

Clinical Outcomes of Pulsed Field versus Thermal Ablation for Paroxysmal Atrial Fibrillation: Systematic Review and Meta-Analysis of Randomized Evidence

**Contents**

| **No.** | **Name** | **Page No.** |
| --- | --- | --- |
| **1.** | **Search strategy** | **2** |
| **2.** | **PICO eligibility criteria** | **3** |
| **3.** | **Full-text reviewed studies** | **4** |
| **4.** | **GRADE evidence profile** | **5** |
| **5.** | **Outcome harmonization framework for all pooled analyses** | **7** |
| **6.** | **Trial-specific efficacy definitions, monitoring, and primary-outcome mapping** | **8** |
| **7.** | **Trial-level mapping and data entered for efficacy outcomes** | **9** |
| **8.** | **Trial-level mapping and data entered for safety and procedural outcomes** | **10** |
| **9.** | **Trial-level PFA platforms, comparator technologies, and procedural workflows** | **12** |
| **10.** | **Detailed PFA catheter systems, pulse characteristics, mapping strategies, and adjunctive-ablation protocols** | **13** |
| **11.** | **Trial-specific rhythm monitoring, follow-up, recurrence ascertainment, and serious adverse-event definitions** | **15** |
| **12.** | **Incremental clinical and methodological contribution relative to prior PFA meta-analyses** | **16** |
| **13.** | **Leave-one-out sensitivity analysis for total procedure time** | **17** |
| **14.** | **Leave-one-out sensitivity analysis for fluoroscopy time** | **18** |
| **15.** | **Leave-one-out sensitivity analysis for left atrial dwell time** | **19** |
| **16.** | **Risk-of-bias assessment** | **20** |

**Supplemental Table S1. Detailed literature searches strategies**

| **Database** | **Search No.** | **Search strategy** |
| --- | --- | --- |
| PubMed | #1 | "Atrial Fibrillation"[MeSH] OR "atrial fibrillation"[Title/Abstract] OR "paroxysmal atrial fibrillation"[Title/Abstract] OR "paroxysmal AF"[Title/Abstract] OR "paroxysmal Afib"[Title/Abstract] OR "AF"[Title/Abstract] OR "Afib"[Title/Abstract] |
| PubMed | #2 | "pulsed field ablation"[Title/Abstract] OR "pulsed-field ablation"[Title/Abstract] OR "pulsed electric field"[Title/Abstract] OR "pulsed electric field ablation"[Title/Abstract] OR "electroporation"[Title/Abstract] OR "irreversible electroporation"[Title/Abstract] OR "PFA"[Title/Abstract] |
| PubMed | #3 | "Catheter Ablation"[MeSH] OR "catheter ablation"[Title/Abstract] OR "pulmonary vein isolation"[Title/Abstract] OR "PVI"[Title/Abstract] OR "radiofrequency ablation"[Title/Abstract] OR "RFA"[Title/Abstract] OR "cryoballoon"[Title/Abstract] OR "cryoballoon ablation"[Title/Abstract] OR "cryoablation"[Title/Abstract] OR "thermal ablation"[Title/Abstract] |
| PubMed | #4 | #1 AND #2 AND #3 |
| Cochrane Library | #1 | MeSH descriptor: [Atrial Fibrillation] explode all trees OR ("atrial fibrillation" OR "paroxysmal atrial fibrillation" OR "paroxysmal AF" OR "paroxysmal Afib" OR AF OR Afib):ti,ab,kw |
| Cochrane Library | #2 | ("pulsed field ablation" OR "pulsed-field ablation" OR "pulsed electric field" OR "pulsed electric field ablation" OR electroporation OR "irreversible electroporation" OR PFA):ti,ab,kw |
| Cochrane Library | #3 | MeSH descriptor: [Catheter Ablation] explode all trees OR ("catheter ablation" OR "pulmonary vein isolation" OR PVI OR "radiofrequency ablation" OR RFA OR cryoballoon OR "cryoballoon ablation" OR cryoablation OR "thermal ablation"):ti,ab,kw |
| Cochrane Library | #4 | #1 AND #2 AND #3 |

**Abbreviations:** AF = atrial fibrillation; CBA = cryoballoon ablation; MeSH = Medical Subject Headings; PFA = pulsed field ablation; PVI = pulmonary vein isolation; RFA = radiofrequency ablation.

**Supplemental Table S2. PICO eligibility criteria**

| **Category** | **Inclusion criteria** | **Exclusion criteria** |
| --- | --- | --- |
| **Population (P)** | • Adults with documented symptomatic paroxysmal atrial fibrillation (AF). • Patients undergoing first catheter ablation with pulmonary vein isolation (PVI) as the intended rhythm-control procedure. • Patients eligible for catheter-based AF ablation according to trial-specific criteria and contemporary guideline indications. • Trial populations randomized to pulsed field ablation (PFA) or conventional thermal ablation. | • Persistent AF, long-standing persistent AF, or non-paroxysmal AF populations unless paroxysmal AF data are separately extractable. • Prior left atrial ablation, prior AF surgery, or repeat-ablation cohorts when first-ablation data are not separable. • AF caused by reversible or non-cardiac secondary causes if not part of the target paroxysmal AF population. • Wrong population, non-AF population, or mixed AF populations without extractable paroxysmal AF data. • Duplicate, overlapping, subanalysis, or follow-up reports of an already included randomized trial when inclusion would double-count participants. |
| **Intervention (I)** | • Pulsed field ablation used as the randomized ablation strategy for PVI. • Eligible PFA technologies included single-shot or focal/multielectrode PFA platforms, including pentaspline PFA and nanosecond/biphasic PFA systems. • Adjunctive cavotricuspid isthmus (CTI) ablation was permitted when clinically indicated or protocol-allowed. | • Non-PFA ablation strategies in the intervention arm. • Single-arm PFA studies without a randomized thermal ablation comparator. • Hybrid or dual-energy strategies in which the independent effect of PFA could not be separated. • Studies evaluating PFA for non-PVI targets or non-paroxysmal AF populations without extractable eligible data. |
| **Comparator (C)** | • Conventional thermal ablation for PVI. • Eligible thermal comparators included radiofrequency ablation (RFA), ablation index-guided RFA, CLOSE-protocol RFA, cryoballoon ablation (CBA), or a conventional thermal control arm containing RFA and/or CBA. | • Medical therapy alone, sham procedures, or non-ablation control groups. • Comparisons between PFA platforms without a thermal ablation comparator. • Thermal-versus-thermal comparisons without PFA. • Non-randomized historical or registry controls. |
| **Outcomes (O)** | • Efficacy outcomes: 12-month treatment success after the blanking period, recurrent atrial arrhythmias, repeat catheter ablation, antiarrhythmic drug resumption after blanking, and electrical cardioversion after blanking. • Safety outcomes: composite serious adverse events or procedure-related serious complications, all-cause death, stroke or transient ischemic attack, cardiac tamponade or perforation, and pulmonary vein stenosis. • Procedural outcomes: total procedure time, left atrial dwell time, fluoroscopy time, CTI line creation, and general anesthesia use. • Outcomes had to provide extractable comparative data as event counts, means with standard deviations, effect estimates, or values mathematically derivable from the report. | • Studies without extractable comparative outcome data for prespecified efficacy, safety, or procedural endpoints. • Endpoint definitions or effect metrics that were not sufficiently comparable and could not be harmonized. • Abstract-only records, conference materials, protocols, or short reports lacking sufficient full-text outcome data. • Cost-only or periprocedural-only reports without eligible comparative clinical follow-up for the main synthesis. |
| **Study design (S)** | • Prospective randomized controlled trials comparing PFA with thermal ablation. • Parallel-group randomized trial designs were eligible. • Full-text peer-reviewed trial reports from unique randomized populations were prioritized for quantitative synthesis. | • Observational studies, registries, retrospective analyses, single-arm studies, case reports, case series, editorials, letters, reviews, prior meta-analyses, and narrative reviews. • Study protocols or trial registry records without completed outcome data. • Conference abstracts or presentations without sufficient peer-reviewed full-text data. • Secondary analyses, subgroup analyses, post hoc analyses, and long-term extensions of included trials when not representing a distinct eligible randomized comparison. |

**Abbreviations:** AF = atrial fibrillation; CBA = cryoballoon ablation; CI = confidence interval; CTI = cavotricuspid isthmus; PFA = pulsed field ablation; PICO = Population, Intervention, Comparator, Outcomes; PVI = pulmonary vein isolation; RCT = randomized controlled trial; RFA = radiofrequency ablation; TIA = transient ischemic attack.

**Supplemental Table S3. Full-text reviewed studies**

| **Author/Year** | **Journal/Source** | **Title / Report** | **Reason for Exclusion** | **Detailed rationale** |
| --- | --- | --- | --- | --- |
| Chierchia et al., 2024 | ESC presentation / industry slide deck | Pulsed Electric Field versus Cryoballoon to Treat Paroxysmal Atrial Fibrillation (PERFECT-PAF) Randomized Trial: A Periprocedural Clinical and Cost Analysis | Conference abstract / short-term periprocedural report | Excluded because the report focused on periprocedural safety, efficiency, and 30-day economic outcomes rather than eligible 12-month efficacy and safety outcomes for the main quantitative synthesis. |
| Reddy et al., 2026 | Nature Medicine | Pulsed field ablation versus conventional thermal ablation for paroxysmal atrial fibrillation: 4-year outcomes in the ADVENT-LTO study | Overlapping population / long-term extension | Excluded because this was an extended follow-up report of the ADVENT trial population and was not an independent randomized trial for the prespecified 12-month outcomes used in the main analysis. |
| Mountantonakis et al., 2025 | Heart Rhythm | Predictors of atrial fibrillation freedom postablation with the pentaspline pulsed field ablation catheter: Subanalysis of the ADVENT Study | Overlapping population / subanalysis | Excluded because this was a secondary/subgroup analysis of the already included ADVENT trial and did not provide an independent randomized comparison for pooling. |

**Supplemental Table S4. GRADE evidence profile for pulsed field ablation versus thermal ablation in paroxysmal atrial fibrillation**

| **Outcome** | **No. of trials** | **Study design** | **Risk of bias** | **Inconsistency** | **Indirectness** | **Imprecision** | **Other considerations** | **Patients: PFA** | **Patients: Thermal** | **Effect: Relative (95% CI)** | **Effect: Absolute (95% CI)** | **Certainty** | **Importance** |
| --- | --- | --- | --- | --- | --- | --- | --- | --- | --- | --- | --- | --- | --- |
| **Efficacy outcomes** |  |  |  |  |  |  |  |  |  |  |  |  |  |
| **12-month treatment success** | 4 | Randomized trials | Seriousᵃ | Not serious | Not serious | Seriousᵇ | None | 475/697 (68.1%) | 450/695 (64.7%) | RR 1.04 (0.97 to 1.12) | 26 more per 1,000 (from 19 fewer to 78 more) | ⨁⨁◯◯ Low | CRITICAL |
| **Repeat catheter ablation** | 4 | Randomized trials | Not serious | Not serious | Not serious | Seriousᵇ | None | 51/696 (7.3%) | 43/693 (6.2%) | RR 1.22 (0.76 to 1.97) | 14 more per 1,000 (from 15 fewer to 60 more) | ⨁⨁⨁◯ Moderate | IMPORTANT |
| **Antiarrhythmic drug resumption** | 4 | Randomized trials | Seriousᵃ | Not serious | Not serious | Seriousᵇ | None | 68/696 (9.8%) | 79/693 (11.4%) | RR 0.86 (0.64 to 1.16) | 16 fewer per 1,000 (from 41 fewer to 18 more) | ⨁⨁◯◯ Low | IMPORTANT |
| **Electrical cardioversion** | 3 | Randomized trials | Seriousᵃ | Not serious | Not serious | Very seriousᶜ | None | 4/555 (0.7%) | 5/551 (0.9%) | RR 0.79 (0.21 to 2.90) | 2 fewer per 1,000 (from 7 fewer to 17 more) | ⨁◯◯◯ Very low | IMPORTANT |
| **Recurrent atrial arrhythmias** | 4 | Randomized trials | Seriousᵃ | Not serious | Not serious | Seriousᵇ | None | 150/696 (21.6%) | 150/693 (21.6%) | RR 1.00 (0.77 to 1.29) | 0 fewer per 1,000 (from 50 fewer to 63 more) | ⨁⨁◯◯ Low | IMPORTANT |
| **Safety outcomes** |  |  |  |  |  |  |  |  |  |  |  |  |  |
| **Composite serious adverse events** | 3 | Randomized trials | Seriousᵃ | Not serious | Not serious | Seriousᵇ | None | 12/555 (2.2%) | 17/551 (3.1%) | RR 0.71 (0.32 to 1.57) | 9 fewer per 1,000 (from 21 fewer to 18 more) | ⨁⨁◯◯ Low | CRITICAL |
| **All-cause death** | 2 | Randomized trials | Seriousᵃ | Not serious | Not serious | Very seriousᶜ | None | 2/410 (0.5%) | 0/407 (0.0%) | RR 2.99 (0.31 to 28.54) | Not reliably estimable because no deaths occurred in the thermal group | ⨁◯◯◯ Very low | CRITICAL |
| **Stroke or transient ischemic attack** | 4 | Randomized trials | Seriousᵃ | Not serious | Not serious | Very seriousᶜ | None | 4/697 (0.6%) | 3/696 (0.4%) | RR 1.21 (0.29 to 4.94) | 1 more per 1,000 (from 3 fewer to 17 more) | ⨁◯◯◯ Very low | CRITICAL |
| **Cardiac tamponade or perforation** | 3 | Randomized trials | Seriousᵃ | Not serious | Not serious | Very seriousᶜ | None | 2/555 (0.4%) | 4/551 (0.7%) | RR 0.58 (0.07 to 4.74) | 3 fewer per 1,000 (from 7 fewer to 27 more) | ⨁◯◯◯ Very low | CRITICAL |
| **Pulmonary vein stenosis** | 2 | Randomized trials | Not serious | Not serious | Not serious | Very seriousᶜ | None | 0/287 (0.0%) | 3/289 (1.0%) | RR 0.26 (0.03 to 2.31) | 8 fewer per 1,000 (from 10 fewer to 14 more) | ⨁⨁◯◯ Low | CRITICAL |
| **Procedural outcomes** |  |  |  |  |  |  |  |  |  |  |  |  |  |
| **Total procedure time** | 4 | Randomized trials | Seriousᵃ | Very seriousᵈ | Not serious | Not serious | None | 697 | 696 | Not applicable | MD -22.42 min (-33.44 to -11.39) | ⨁◯◯◯ Very low | IMPORTANT |
| **Left atrial dwell time** | 4 | Randomized trials | Seriousᵃ | Very seriousᵈ | Not serious | Not serious | None | 697 | 696 | Not applicable | MD -25.73 min (-34.66 to -16.79) | ⨁◯◯◯ Very low | IMPORTANT |
| **Fluoroscopy time** | 4 | Randomized trials | Seriousᵃ | Very seriousᵈ | Not serious | Not serious | None | 697 | 696 | Not applicable | MD 7.34 min (0.39 to 14.30) | ⨁◯◯◯ Very low | IMPORTANT |
| **Cavotricuspid isthmus line creation** | 4 | Randomized trials | Seriousᵃ | Not serious | Not serious | Seriousᵇ | None | 94/697 (13.5%) | 122/696 (17.5%) | RR 0.75 (0.51 to 1.09) | 44 fewer per 1,000 (from 86 fewer to 16 more) | ⨁⨁◯◯ Low | IMPORTANT |
| **General anesthesia use** | 4 | Randomized trials | Seriousᵃ | Not serious | Not serious | Not serious | Practice-pattern/process measure; limited clinical relevanceᶠ | 357/697 (51.2%) | 370/696 (53.2%) | RR 0.94 (0.91 to 0.98) | 32 fewer per 1,000 (from 48 fewer to 11 fewer) | ⨁⨁⨁◯ Moderate | LIMITED IMPORTANCE |

**Footnotes**

ᵃ Downgraded one level for risk of bias because one or more contributing randomized trials had some concerns, particularly ADVENT 2023 in outcomes where it contributed meaningful weight.

ᵇ Downgraded one level for imprecision because the 95% confidence interval crossed the line of no effect and included clinically important benefit and harm.

ᶜ Downgraded two levels for very serious imprecision because event rates were very low and the optimal information size was not met.

ᵈ Downgraded two levels for very serious inconsistency because heterogeneity was substantial to considerable across procedural analyses.

ᶠ General anesthesia use was treated as a procedural-context/process measure. The certainty rating reflects confidence in the observed association and does not imply patient-centered clinical importance or an energy-specific advantage.

**Abbreviations:** AF = atrial fibrillation; CI = confidence interval; CTI = cavotricuspid isthmus; GRADE = Grading of Recommendations Assessment, Development and Evaluation; LA = left atrial; MD = mean difference; PFA = pulsed field ablation; RR = risk ratio; TIA = transient ischemic attack.

**Supplemental Table S5A. Outcome harmonization framework applied before quantitative pooling**

| **Decision step** | **Rule applied** | **Rationale and implementation** |
| --- | --- | --- |
| **Prespecification and timing** | The review outcome domains, direction of effect, and approximately 12-month follow-up were prespecified. Detailed cross-trial mapping rules were finalized during data extraction and before quantitative pooling. | This distinction avoids claiming that every trial-specific operational rule appeared verbatim in the registry while confirming that mapping was completed before effect estimates were combined. |
| **Independent application** | Two reviewers independently mapped each reported endpoint to the prespecified outcome domains; disagreements were resolved by consensus and third-reviewer adjudication. | Reduced subjective post hoc selection of favorable definitions. |
| **Preferred analysis population** | Intention-to-treat, modified intention-to-treat, or full-analysis-set data were preferred, according to the original trial report. | Preserved randomized comparisons and the trial-specific handling of crossovers, missing outcomes, and censored observations. No participant-level re-imputation was performed by the review team. |
| **Primary efficacy direction** | A favorable binary event was defined as treatment success through approximately 12 months. Trial-defined primary success endpoints were used directly. When a trial reported recurrence as the primary endpoint, freedom from recurrence was calculated as the complement of the directly reported recurrence count. | Established a common event direction without changing the underlying trial event definition. |
| **Composite efficacy endpoints** | Original composite components were retained. Components were not selectively removed, added, or reconstructed to create a new composite. | Avoided outcome redefinition and selective component handling. |
| **Secondary efficacy outcomes** | Recurrent atrial arrhythmia, repeat ablation, antiarrhythmic drug resumption, and electrical cardioversion were pooled only when directly reported or derivable without redefining the event. Trial-specific blanking periods and reporting windows were retained. | Preserved the original trial definitions while making cross-trial differences transparent. Component outcomes could overlap within a participant and were analyzed separately; they were never summed to reconstruct treatment failure. |
| **Safety composite** | Trial-defined serious device/procedure-related or serious procedural complication composites were pooled only when the clinical construct and time window were sufficiently comparable. Broad all-cause adverse-event composites were not forced into this analysis. | Reduced clinical misclassification caused by materially different safety composites. |
| **Individual safety outcomes** | Death, stroke/TIA, tamponade/perforation, and pulmonary vein stenosis were retained as trial-reported patient-level events. Trial-specific thresholds and ascertainment strategies were documented. | Rare-event estimates reflect different surveillance intensity and should not be interpreted as proof of equivalence. |
| **Double-zero studies** | A study reporting zero events in both randomized arms was documented in the mapping table but excluded from the relative-risk calculation for that outcome. | Such a study contains no information about the relative effect under the Mantel-Haenszel risk-ratio model. |
| **Continuous procedural outcomes** | Trial-reported means and standard deviations were pooled in minutes. Original trial start/stop definitions were retained; no attempt was made to impose an unreported common operational definition. | Avoided unsupported transformation while acknowledging clinical heterogeneity in procedural workflow. |
| **Binary procedural outcomes** | Cavotricuspid isthmus ablation and general anesthesia use were pooled as the reported number of participants with the event. | Cavotricuspid isthmus ablation was retained as a procedural outcome. General anesthesia use was interpreted as a practice-pattern/process measure rather than a patient-centered clinical benefit or an intrinsic energy-specific effect. |
| **Mixed thermal comparator** | The ADVENT radiofrequency/cryoballoon control was retained as the single randomized thermal arm reported by the trial. | Prevented double-counting of the shared PFA group and preserved the randomized trial comparison. |
| **Units and transformations** | No unit conversion was required for time outcomes. The only direction transformation was recurrence to freedom from recurrence for the SINGLE SHOT CHAMPION contribution to the primary favorable-event analysis. | Made all transformations explicit and reproducible. |

**Note:** Outcome domains were prespecified in the registered review protocol; the detailed mapping rules above describe the pre-pooling implementation used for the present synthesis. Trial-level sources correspond to references 10-13 in the main manuscript.

**Supplemental Table S5B. Trial-specific efficacy definitions, rhythm monitoring, and mapping to the pooled primary treatment-success outcome**

| **Trial** | **Blanking period** | **Original primary efficacy endpoint** | **Rhythm monitoring and recurrence ascertainment** | **Analysis population and missing-data handling** | **Events/total entered as treatment success** | **Rationale for selection and key limitation** |
| --- | --- | --- | --- | --- | --- | --- |
| **ADVENT** | 3 months | Freedom through 1 year from a composite of initial failure to achieve PVI using the assigned modality, AF/AFL/AT lasting >=30 s after the blanking period, class I/III AAD use or cardioversion after the blanking period, amiodarone use at any time, or repeat ablation at any time. | Telephone contacts at 7, 30, and 60 days; visits at 3, 6, and 12 months; 72-h Holter monitoring at 6 and 12 months; weekly transtelephonic ECG after the blanking period and symptom-triggered recordings. Events were adjudicated. | Modified intention-to-treat population: randomized participants who had a trial ablation catheter inserted and received energy (305 PFA; 302 thermal). The trial used Bayesian multiple imputation for censored primary-outcome observations. | 204/305 PFA; 194/302 thermal | Used directly because this was the trial-defined primary binary efficacy-success endpoint at 1 year. Limitation: broad composite and intermittent rhythm surveillance; the thermal arm combined RFA and cryoballoon according to site assignment. |
| **SINGLE SHOT CHAMPION** | 90 days | First recurrence of AF/AFL/AT from day 91 through day 365. A device-detected episode had to last >=120 s and contain >=30 s of continuous atrial tachyarrhythmia. | Continuous implantable cardiac monitoring in all participants, daily automated transmissions to a core laboratory, blinded adjudication, and visits at 3, 6, and 12 months. | Intention-to-treat population: 105 PFA and 105 cryoballoon participants. Time-to-event analyses accounted for censoring; the directly reported patient recurrence counts were used for the binary mapping. | 66/105 PFA; 52/105 cryoballoon, calculated as 105 minus the reported primary recurrences of 39 and 53, respectively | Selected as the closest trial-defined approximately 12-month favorable rhythm outcome. Limitation: it was a recurrence-only endpoint detected by continuous monitoring and did not include AAD use, cardioversion, repeat ablation, or initial procedural failure in the primary construct. |
| **BEAT PAROX-AF** | 60 days | 12-month single-procedure clinical success: successful index ablation, no AF/AFL/AT recurrence >=30 s on any recording after blanking, no class I/III AAD resumption after blanking, no cardioversion after blanking, and no redo ablation at any time except for typical flutter. | Telephone contact at 1 month; visits at 2, 6, and 12 months with 12-lead ECG and 24-h Holter; weekly Kardia single-lead ECG; symptom-triggered recordings; available telemetry. Recurrences were adjudicated by a blinded committee. | Primary intention-to-treat analysis: 145 PFA and 144 RFA. The primary endpoint was available for 288/289 participants; the published binary result was 112/145 versus 111/143. Multiple imputation was used in the adjusted trial analysis. | 112/145 PFA; 111/143 RFA | Used directly because this was the trial-defined primary binary success endpoint at 12 months. Limitation: shorter blanking period and intensive, but intermittent, ECG surveillance. |
| **InsightPFA** | 3 months | Freedom during the 9-month assessment period from documented AF/AFL/AT >=30 s without class I/III AAD therapy; after blanking, documented recurrence, class I/III AAD use including amiodarone, cardioversion, or repeat ablation constituted failure, with additional trial-specific amiodarone rules. | On-site follow-up at 1, 3, 6, and 12 months; 12-lead ECG and 24-h Holter at each visit; prompt ECG for arrhythmia-related symptoms. No continuous monitor was used. | Full analysis set: 142 nsPFA and 145 AI-guided RFA. The published primary FAS analysis treated the one nsPFA and three RFA participants with missing primary outcome data as failures in the main reported estimate. | 93/142 nsPFA; 93/145 AI-guided RFA | Used directly because this was the trial-defined binary primary success endpoint. Limitation: intermittent monitoring and trial-specific treatment-failure rules. |

**Interpretation:** The pooled primary analysis estimates the comparative treatment effect across clinically aligned trial-specific efficacy constructs. It should not be interpreted as a pooled absolute success rate under one uniform endpoint or monitoring protocol.

**Abbreviations:** AAD = antiarrhythmic drug; AF = atrial fibrillation; AFL = atrial flutter; AT = atrial tachycardia; ECG = electrocardiogram; FAS = full analysis set; PFA = pulsed field ablation; PVI = pulmonary vein isolation; RFA = radiofrequency ablation.

**Supplemental Table S5C. Trial-level mapping and exact data entered for all pooled efficacy outcomes**

| **Pooled outcome** | **ADVENT** | **SINGLE SHOT CHAMPION** | **BEAT PAROX-AF** | **InsightPFA** | **Harmonization decision and limitation** |
| --- | --- | --- | --- | --- | --- |
| **12-month treatment success (favorable event)** | 204/305 PFA vs 194/302 thermal; trial primary composite success at 1 year. | 66/105 PFA vs 52/105 cryoballoon; freedom from primary recurrence, calculated as the complement of 39/105 and 53/105 recurrences. | 112/145 PFA vs 111/143 RFA; trial primary single-procedure success at 12 months. | 93/142 nsPFA vs 93/145 RFA; trial primary FAS success with trial-specified missing-data handling. | Mapped to a common favorable direction. Composite components and trial-specific monitoring were retained; definitions were not treated as identical. |
| **Repeat catheter ablation (unfavorable event)** | 14/305 vs 20/302; participants undergoing repeat ablation for clinical recurrence through 1 year. These total repeat-procedure counts, rather than the hierarchical reason-for-primary-failure counts, were used. | 16/105 vs 10/105; secondary clinical endpoint of repeat ablation during 12-month follow-up. | 13/145 vs 9/144; repeat ablation at any time, with the primary composite excluding redo solely for typical flutter. | 8/141 vs 4/142; surgery or catheter ablation for AF/AFL/AT after the blanking period. | Direct patient-level repeat-ablation counts were pooled. Trial-specific indication and timing rules were retained. |
| **Antiarrhythmic drug resumption (unfavorable event)** | 24/305 vs 27/302; class I/III AAD use after the 3-month blanking period. Amiodarone-at-any-time was a separate primary-composite component and was not added to this component analysis. | 3/105 vs 3/105; reinitiation of antiarrhythmic medication during 12-month follow-up after protocol-mandated discontinuation following blanking. | 9/145 vs 12/144; class I/III AAD resumption after the 60-day blanking period. | 32/141 vs 37/142; class I/III AAD use after the 3-month blanking period. | Direct trial-reported AAD-resumption counts were pooled; drug-class and timing rules were retained. |
| **Electrical cardioversion (unfavorable event)** | 1/305 vs 0/302; cardioversion after the 3-month blanking period. | 2/105 vs 4/105; electrical cardioversion for atrial-arrhythmia recurrence during 12-month follow-up; a post-blanking-only count was not separately reported. | 1/145 vs 1/144; cardioversion after the 60-day blanking period. | Not included: cardioversion was part of the treatment-failure construct but a separate comparative patient count was not reported in the extractable primary publication/supplement. | Only directly extractable patient-level counts were pooled. The SINGLE SHOT CHAMPION reporting window was retained and is a source of clinical heterogeneity. |
| **Recurrent atrial arrhythmia (unfavorable event)** | 51/305 vs 48/302; AF/AFL/AT >=30 s after the 3-month blanking period, detected by scheduled and symptom-triggered intermittent monitoring. | 39/105 vs 53/105; first primary recurrence from day 91 to 365, detected continuously by implantable monitor. | 27/145 vs 23/144; AF/AFL/AT >=30 s after the 60-day blanking period on any available recording, including weekly Kardia and scheduled Holter. | 33/141 vs 26/142; ECG/AECG-documented AF/AFL/AT >=30 s after the 3-month blanking period. | All estimates used trial-defined post-blanking recurrence as an unfavorable event. No threshold or surveillance-intensity standardization was imposed; monitoring differences limit comparison of absolute recurrence rates. |

**Data convention:** Values are events/total and correspond to the study-level entries in Figure 2A-E. For Figure 2A, an event denotes treatment success; for Figures 2B-E, an event denotes the named unfavorable outcome. Component events may overlap within the same participant and were analyzed separately.

**Abbreviations:** AAD = antiarrhythmic drug; AECG = ambulatory electrocardiography; AF = atrial fibrillation; AFL = atrial flutter; AT = atrial tachycardia; FAS = full analysis set; PFA = pulsed field ablation; RFA = radiofrequency ablation.

**Supplemental Table S5D. Trial-level mapping and exact data entered for all pooled safety and procedural outcomes**

| **Pooled outcome** | **ADVENT** | **SINGLE SHOT CHAMPION** | **BEAT PAROX-AF** | **InsightPFA** | **Harmonization decision and limitation** |
| --- | --- | --- | --- | --- | --- |
| **Composite serious adverse events** | 6/305 vs 4/302; prespecified device/procedure-related SAEs within 7 days; atrioesophageal fistula and PV stenosis counted regardless of timing. | 1/105 vs 2/105; 30-day composite of tamponade requiring drainage, persistent phrenic palsy >24 h, serious vascular complication requiring intervention, stroke/TIA, atrioesophageal fistula, or death. | 5/145 vs 11/144; prespecified device/procedure-related SAEs within 7 days; atrioesophageal fistula and PV stenosis counted regardless of timing. | Not pooled: the trial reported a broader safety framework (procedure-related complications, device-related events, all AEs and SAEs) without one directly comparable composite count. | Only sufficiently comparable trial-defined serious procedural/device composites were pooled. Definitions and time windows remained nonidentical, so the estimate does not establish equivalence. |
| **All-cause death** | 1/305 vs 0/302. | 1/105 vs 0/105; death from cancer at 11 months. | 0/145 vs 0/144; documented but excluded from RR model as double-zero. | 0/142 vs 0/145; documented but excluded from RR model as double-zero. | All-cause death was used when separately reported. Double-zero trials contributed no relative-effect information. |
| **Stroke or transient ischemic attack** | 1/305 vs 1/302; one TIA with PFA and one stroke with thermal ablation in the device/procedure-related event table. | 1/105 vs 2/105 through 12 months. | 1/145 vs 0/144; one TIA with PFA. | 1/142 vs 0/145; one cerebral infarction with nsPFA. | Stroke and TIA were combined as a patient-level binary outcome. Trial-specific event windows and relatedness classifications were retained. |
| **Cardiac tamponade or perforation** | 2/305 vs 0/302; device/procedure-related events. | 0/105 vs 2/105; tamponade requiring drainage within the safety window. | 0/145 vs 2/144; procedure-related events. | 0/142 vs 0/145; documented but excluded from RR model as double-zero. | Trial-reported tamponade/perforation was pooled. Event definitions were clinically aligned but time windows differed. |
| **Pulmonary vein stenosis** | 0/305 vs 0/302; routine 3-month CT/MRI assessment; excluded from RR model as double-zero. | Not included: no separately extractable eligible PV-stenosis endpoint was reported for quantitative pooling. | 0/145 vs 2/144; >=70% stenosis, with routine CT/MRI screening at 2 months. | 0/142 vs 1/145; one mild left inferior PV narrowing detected by symptom-triggered CT at 3 months; no routine imaging for all participants. | Trial-reported PV stenosis was pooled without redefining severity thresholds. Different imaging intensity and thresholds materially limit comparison of absolute rates. |
| **Total procedure time, min** | 105.8 +/- 29.4 vs 123.1 +/- 42.1; venous access to access closure, including the required 20-min post-ablation wait and CTI ablation time. | 54.8 +/- 22.7 vs 73.2 +/- 26.7; trial-reported procedure duration; no more specific common start/stop definition was imposed. | 59 +/- 22 vs 98 +/- 33; trial-reported total procedure duration. | 103.99 +/- 33.44 vs 118.77 +/- 32.96; first sheath insertion to final sheath removal. | Means and SDs were pooled in minutes. Different operational definitions and workflows were retained and contribute to heterogeneity. |
| **Left atrial dwell time, min** | 59.4 +/- 18.3 vs 83.7 +/- 30.3; time the ablation catheter was in the left atrium. | 36.1 +/- 16.6 vs 51.5 +/- 20.0; trial-reported LA dwell/indwelling time. | 39 +/- 16 vs 77 +/- 28; trial-reported LA dwell time. | 60.16 +/- 27.07 vs 85.51 +/- 27.21; first entry of the ablation catheter into the LA to complete removal from the LA. | Means and SDs were pooled in minutes. Catheter workflow and trial-specific start/stop rules were retained. |
| **Fluoroscopy time, min** | 21.1 +/- 11.0 vs 13.9 +/- 12.8. | 14.6 +/- 7.2 vs 15.1 +/- 7.9. | 12 +/- 6 vs 7 +/- 6. | 24.63 +/- 9.69 vs 6.96 +/- 5.97. | Trial-reported means and SDs were pooled in minutes. Mapping strategy, device workflow, and operator practice were not standardized. |
| **Cavotricuspid isthmus ablation** | 70/305 vs 86/302. | 14/105 vs 12/105; RF CTI ablation for documented typical flutter with bidirectional block as the endpoint. | 6/145 vs 14/144; RF CTI ablation for typical flutter; bidirectional block achieved. | 4/142 vs 10/145; RF CTI block required for documented or spontaneous typical flutter. | Pooled as the number of participants receiving CTI ablation. Indication and protocol were retained; this is a procedural-context variable. |
| **General anesthesia use** | 277/305 vs 291/302. | 5/105 vs 7/105; most procedures used deep sedation. | 60/145 vs 61/144; GA was introduced at two centers for a substudy rather than mandated across the protocol. | 15/142 vs 11/145; anesthesia selection was unrestricted. | Pooled as observed GA use; the crude absolute difference was 1.9 percentage points lower with PFA (51.2% vs 53.2%). Interpreted as a context-dependent process measure, not a patient-centered or energy-specific advantage. |

**Data convention:** Binary values are events/total and continuous values are mean +/- SD, matching Figures 3A-E and 4A-E. A double-zero study was documented but excluded from the Mantel-Haenszel risk-ratio estimate for that outcome. "Not included" indicates that a separately extractable endpoint meeting the meta-analysis construct was unavailable.

**Abbreviations:** AE = adverse event; CT = computed tomography; CTI = cavotricuspid isthmus; GA = general anesthesia; LA = left atrial; MRI = magnetic resonance imaging; PFA = pulsed field ablation; PV = pulmonary vein; RF = radiofrequency; RR = risk ratio; SAE = serious adverse event; TIA = transient ischemic attack.

**Sources:** Original randomized trial reports and their supplementary appendices corresponding to references 10-13 in the main manuscript.

**Supplemental Table S6. Trial-level PFA platforms, comparator technologies, and procedural workflows**

This table summarizes the major technological and workflow differences that limit interpretation of the pooled procedural estimates as uniform energy-specific effects.

| **Trial** | **PFA platform and energy delivery** | **PFA mapping, verification, and adjunctive workflow** | **Thermal comparator and workflow** | **Anesthesia / procedural context** | **Implication for pooled interpretation** |
| --- | --- | --- | --- | --- | --- |
| **ADVENT (10)** | FARAWAVE multielectrode pentaspline catheter; microsecond-scale, high-voltage PFA delivered over a guidewire through a deflectable sheath. | PVI confirmed by entrance block after a mandatory 20-minute observation period. ICE was used; postablation electroanatomic mapping was optional. RF could be used for clinically manifested non-PV arrhythmias, but empiric lesion sets were not permitted. | Site-specific mixed thermal control: saline-irrigated contact-force RFA with an electroanatomic mapping system (typically 25-50 W) or 23/28-mm cryoballoon ablation with 2-4-minute applications. | General anesthesia or deep sedation was selected at operator discretion; GA was used in 277/305 (90.8%) PFA and 291/302 (96.4%) thermal procedures. | The control arm combined point-by-point RFA and single-shot cryoballoon workflows; therefore, the pooled ADVENT contrast is not modality specific. |
| **SINGLE SHOT CHAMPION (11)** | FARAWAVE multielectrode pentaspline PFA system; single-shot PVI workflow. | Trial-specific single-shot PFA workflow. The primary trial comparison focused on PFA versus cryoballoon rather than a point-by-point mapped RFA strategy. | Arctic Front cryoballoon ablation; single-shot balloon-based PVI. | Deep sedation with midazolam, fentanyl, and propofol was routine; GA was reserved for patients at higher risk from sedation and was used in 5/105 (4.8%) PFA and 7/105 (6.7%) cryoballoon procedures. | This was a single-shot versus single-shot comparison and should not be used to infer the relative efficiency of PFA versus point-by-point RFA. |
| **BEAT PAROX-AF (12)** | FARAPULSE pentaspline system; 2,000-V high-voltage PFA with at least 8 applications per PV in basket and flower configurations. | Single-shot PFA workflow; procedural comparison was performed in high-volume centers experienced with both strategies. | Point-by-point contact-force RFA using the standardized CLOSE protocol, CARTO electroanatomic mapping, and a SMARTTOUCH catheter. | The initial protocol used conscious sedation with benzodiazepines, morphine, and propofol; GA was later introduced at two Czech centers for an autonomic-nervous-system substudy and was used in 60/145 (41.4%) PFA and 61/144 (42.4%) RFA procedures. | The comparator was an optimized, reproducible mapped RFA strategy rather than generic 'thermal ablation'; procedure-time differences are specific to this benchmark. |
| **InsightPFA (13)** | LotosPFA/InRythm nanosecond system; unsynchronized biphasic waveform at 2,100 V. Four spindle applications were delivered at the PV ostium and four lotus applications at the antrum. | A 20-minute observation period was required. Three-dimensional mapping was optional in the PFA arm and was used in 54.2% of patients; supplemental ablation was delivered for recovered PV conduction. | AI-guided RFA using CARTO, Pentaray or LASSO mapping, and THERMOCOOL SMARTTOUCH; target AI 400-450 anteriorly and 300-350 posteriorly. | Conscious sedation or GA was selected at investigator discretion; conscious sedation was used in 127/142 (89.4%) nsPFA and 134/145 (92.4%) RFA procedures, corresponding to GA use in 15/142 and 11/145, respectively. | This trial evaluated a technologically distinct nanosecond platform and an asymmetric mapping workflow; its procedural effects should not be generalized to microsecond pentaspline PFA. |

Abbreviations: AI = ablation index; CARTO = electroanatomic mapping system; GA = general anesthesia; ICE = intracardiac echocardiography; PFA = pulsed field ablation; PV = pulmonary vein; PVI = pulmonary vein isolation; RF/RFA = radiofrequency ablation. Interpretive note: pooled estimates in the meta-analysis are average class-level contrasts across these trial-specific configurations and do not establish interchangeability among PFA platforms or thermal comparator modalities.

**Supplemental Table S7. Trial-specific PFA platforms, pulse-delivery characteristics, mapping strategies, and adjunctive ablation protocols**

Platform details were extracted from the original trial reports and supplementary appendices. “Not reported” indicates that the publication did not provide a more granular waveform or pulse specification. These data are descriptive and are not intended to support cross-trial platform comparisons.

| **Trial** | **PFA catheter / system** | **Waveform and pulse characteristics (as reported)** | **Application / lesion-delivery protocol** | **Mapping, waiting period, and PVI verification** | **Supplemental or adjunctive ablation protocol** | **Comparator / anesthesia context and interpretive limitation** |
| --- | --- | --- | --- | --- | --- | --- |
| **ADVENT (10)** | FARAWAVE 31- or 35-mm multielectrode pentaspline catheter, delivered over a guidewire through a 13-F FARADRIVE deflectable sheath; FARASTAR generator. | Microsecond-scale, high-voltage PFA. Each application consisted of 5 pulse packets delivered over 2.5 seconds, was not QRS-gated, and used 1,800, 1,900, or 2,000 V. | Nominally 8 applications per PV: 4 in basket configuration and 4 in flower configuration, with catheter rotation between pairs of applications. Additional applications were permitted at operator discretion. | Baseline and postablation electroanatomic mapping were optional. ICE was used during PFA. Entrance block was confirmed after a mandatory 20-minute waiting period; recovered conduction prompted additional PFA. | Conventional RF was permitted for documented or induced typical flutter, accessory pathway, AV nodal re-entrant tachycardia, treatment-emergent left atrial flutter, or incessant atrial tachycardia. Empiric non-PV lesion sets were prohibited. | Site-specific mixed comparator: contact-force RFA with electroanatomic mapping (typically 25-50 W) or 23-/28-mm cryoballoon ablation with 2-4-minute applications. GA or deep sedation was selected at operator discretion. Mixed comparator and optional mapping limit modality-specific interpretation. |
| **SINGLE SHOT CHAMPION (11)** | FARAWAVE 31- or 35-mm pentaspline catheter advanced over a J-tip guidewire through a 13-F FARADRIVE sheath; FARAPULSE system. | Bipolar 2.0-kV PFA. Each application consisted of 5 pulse packets delivered over 2.5 seconds and was not QRS-gated; exact pulse width was not reported. | At least 4 applications per PV in basket configuration and 4 in flower configuration (at least 8 per PV and 32 overall); additional applications were permitted at operator discretion. | Acute PVI was assessed at procedure end with the FARAWAVE catheter. No waiting period or adenosine testing was used. In the first 25 patients per arm, an adjunctive 3D-mapping substudy used Orion/Rhythmia after fluoroscopy-guided PVI. | No additional left atrial lesions were permitted. In patients with documented typical right atrial flutter, CTI ablation was performed with RF to bidirectional block in both groups. | Arctic Front 23-/28-mm cryoballoon with a time-to-effect-plus-2-minute strategy; no bonus freezes, waiting period, or adenosine testing. Deep sedation was routine and GA was reserved for high-risk patients. This was a single-shot-versus-single-shot comparison. |
| **BEAT PAROX-AF (12)** | FARAPULSE system with 31- or 35-mm FARAWAVE pentaspline catheter. | High-voltage PFA at 2,000 V was reported. More granular waveform, pulse-width, and packet-structure specifications were not reported in the trial publication or published protocol. | Minimum of 8 applications per PV using basket and flower configurations, with catheter rotation for circumferential ostial and antral coverage; additional applications were permitted at operator discretion. | No electroanatomic mapping system was used in the PFA arm. PVI was confirmed by entrance and/or exit block. No waiting period after PVI was specified in the protocol. | CTI ablation for prior or inducible typical flutter was performed with RF. Accessory pathways, AV nodal re-entrant tachycardia, incidental left atrial flutter, or incessant atrial tachycardia could be treated with RF. No additional atrial lesions were created for index paroxysmal AF. | Point-by-point contact-force RFA using CARTO 3, SMARTTOUCH/SMARTTOUCH Surround Flow, and the CLOSE protocol (35-45 W; interlesion distance <=6 mm; AI 300-400 posteriorly and >=500 anteriorly). Conscious sedation was standard; GA was later introduced at two centers. Findings are specific to an optimized mapped-RFA benchmark. |
| **InsightPFA (13)** | LotosPFA over-the-wire catheter with InRythm system, delivered through a 12-F InBridge steerable sheath. | Nanosecond-scale, biphasic waveform, unsynchronized to cardiac rhythm, at 2,100 V. Each application comprised 5 consecutive pulses; exact pulse width was not reported. | Four applications were delivered at the PV ostium in spindle configuration and 4 at the antrum in lotus configuration at the same voltage; catheter rotation was required. | A mandatory 20-minute observation period followed PVI. Recovered PV conduction prompted supplemental ablation and another 20-minute observation period. Post-PFA 3D electroanatomic mapping was optional and used in 77/142 patients (54.2%). | Supplemental PFA was delivered for recovered PV conduction. For documented or spontaneous typical flutter, CTI block was created with a THERMOCOOL SMARTTOUCH RF catheter. | AI-guided RFA used CARTO with Pentaray or LASSO mapping and THERMOCOOL SMARTTOUCH (AI 400-450 anteriorly and 300-350 posteriorly). Conscious sedation or GA was investigator-selected; 3D mapping was mandatory in RFA but optional in PFA. This was the only nanosecond-platform trial. |

Abbreviations: AI = ablation index; AV = atrioventricular; CARTO = electroanatomic mapping system; CTI = cavotricuspid isthmus; GA = general anesthesia; ICE = intracardiac echocardiography; PFA = pulsed field ablation; PV = pulmonary vein; PVI = pulmonary vein isolation; RF/RFA = radiofrequency ablation. Source: original randomized trial reports and supplementary appendices corresponding to references 10-13 in the main manuscript. Interpretive note: platform-specific subgroup analysis was not performed because only one trial evaluated LotosPFA/InRythm; cross-platform contrasts would be driven by a single trial and confounded by comparator and workflow differences.

**Supplemental Table S8. Trial-specific rhythm monitoring, follow-up, recurrence ascertainment, and serious adverse-event definitions**

This table presents the trial-level surveillance and safety definitions relevant to interpretation of the pooled 12-month treatment-success, recurrent-arrhythmia, and composite serious-adverse-event estimates.

| **Trial** | **Blanking period** | **Scheduled follow-up** | **Rhythm monitoring and recurrence ascertainment** | **Post-blanking recurrence definition** | **Serious adverse-event definition and ascertainment** | **Implication for pooled interpretation** |
| --- | --- | --- | --- | --- | --- | --- |
| **ADVENT (10)** | 3 months | Telephone contacts at 7, 30, and 60 days; study visits at 3, 6, and 12 months. | Weekly transtelephonic ECG after the blanking period; symptom-triggered recordings; 72-hour Holter monitoring at 6 and 12 months. Efficacy events were adjudicated. | Documented AF, AFL, or AT lasting >=30 seconds after blanking. Recurrence was one component of a broader 1-year treatment-success composite that also included initial PVI failure, AAD use, cardioversion, amiodarone use, and repeat ablation. | Prespecified device- or procedure-related SAEs within 7 days after the procedure; atrioesophageal fistula and PV stenosis counted as SAEs regardless of timing. The pooled composite used 6/305 vs 4/302. | Intermittent surveillance may miss asymptomatic episodes. The efficacy construct was composite, and the SAE definition/window was not identical to those of the other contributing trials. |
| **SINGLE SHOT CHAMPION (11)** | 90 days | Clinical visits at 3, 6, and 12 months. | Continuous implantable cardiac monitoring in all participants, with daily automated transmissions to a core laboratory and blinded event adjudication. | First AF, AFL, or AT recurrence from day 91 through day 365. A device-detected episode had to last >=120 seconds and contain >=30 seconds of continuous atrial tachyarrhythmia. | Thirty-day composite of procedure-related complications: tamponade requiring drainage, persistent phrenic palsy >24 hours, serious vascular complication requiring intervention, stroke/TIA, atrioesophageal fistula, or death. The pooled composite used 1/105 vs 2/105. | Continuous surveillance was more sensitive for asymptomatic recurrence than the intermittent strategies used elsewhere; its safety construct and 30-day window also differed. |
| **BEAT PAROX-AF (12)** | 60 days | Telephone contact at 1 month; visits at 2, 6, and 12 months with systematic 12-lead ECG and 24-hour Holter monitoring. | Weekly Kardia single-lead ECG recordings, symptom-triggered recordings, available in-hospital telemetry, scheduled ECG/Holter monitoring, and blinded clinical-event-committee adjudication. | AF, AFL, or AT lasting >=30 seconds on any recording after the 60-day blanking period. Recurrence was part of the 12-month single-procedure clinical-success composite. | Prespecified device- or procedure-related SAEs within 7 days after the procedure; atrioesophageal fistula and PV stenosis classified as SAEs regardless of timing and adjudicated by the clinical events committee. The pooled composite used 5/145 vs 11/144. | Surveillance was intensive but intermittent, the blanking period was shorter, and the composite safety definition was not identical to those of ADVENT or SINGLE SHOT CHAMPION. |
| **InsightPFA (13)** | 3 months | On-site follow-up at 1, 3, 6, and 12 months. | 12-lead ECG and 24-hour Holter monitoring at each visit; prompt ECG for arrhythmia-related symptoms. No continuous implantable monitor was used. | Documented AF, AFL, or AT lasting >=30 seconds after blanking; post-blanking AAD use, cardioversion, or repeat ablation also constituted treatment failure under trial-specific rules. | Broader trial-period safety assessment including death, stroke/TIA, procedure-related complications, device-related AEs/SAEs, and all AEs/SAEs. No single directly comparable serious procedural/device composite was entered into the pooled SAE analysis. | Intermittent monitoring may underdetect asymptomatic episodes relative to continuous surveillance. The broader safety framework was described but excluded from the pooled composite to avoid clinical misclassification. |

Interpretive note: Continuous monitoring can detect more asymptomatic recurrences than intermittent surveillance. The pooled efficacy estimates preserve randomized within-trial contrasts but should not be interpreted as absolute event rates under a uniform monitoring protocol. The pooled composite serious-adverse-event analysis included ADVENT, SINGLE SHOT CHAMPION, and BEAT PAROX-AF only; definitions and ascertainment windows were not identical.

Abbreviations: AAD = antiarrhythmic drug; AE = adverse event; AF = atrial fibrillation; AFL = atrial flutter; AT = atrial tachycardia; ECG = electrocardiogram; PFA = pulsed field ablation; PV = pulmonary vein; PVI = pulmonary vein isolation; SAE = serious adverse event; TIA = transient ischemic attack. Sources: original randomized trial reports and supplementary appendices corresponding to references 10-13 in the main manuscript.

**Supplemental Table S9. Incremental clinical and methodological contribution relative to prior PFA meta-analyses**

This table summarizes differences in scope, trial selection, analytical methods, and interpretation between the present review and earlier PFA meta-analyses.

| **Review** | **Evidence base and population** | **Key methods** | **Principal conclusion** | **Incremental interpretation or remaining limitation** |
| --- | --- | --- | --- | --- |
| **de Campos et al., 2024 (14)** | Search through February 2024; 18 randomized and nonrandomized studies; 4,998 patients with paroxysmal or persistent AF; PFA compared with RFA or CBA. | Mixed-design comparative meta-analysis of efficacy, safety, procedure time, and fluoroscopy time, with subgroup analyses by AF type and thermal comparator; no formal GRADE assessment. | PFA was associated with shorter procedure time, longer fluoroscopy time, lower treatment failure and esophageal injury, and higher tamponade. | Mixed study designs, observational confounding, and substantial procedural heterogeneity limited causal and energy-specific interpretation. |
| **Qamar et al., 2024 (15)** | Search through October 2023; 26 studies and 2,561 patients; 13 observational studies, 12 nonrandomized clinical trials, and 1 RCT; paroxysmal and persistent AF. | Single-arm random-effects pooling of proportions and means for acute PVI, procedure and fluoroscopy times, arrhythmia recurrence, and complications; no thermal comparator or GRADE assessment. | Acute PVI success was 99.7%; post-blanking atrial arrhythmia recurrence was 17.3%; the pooled complication rate was 2.8%. | The predominantly nonrandomized, noncomparative evidence described event rates but could not estimate the relative effect of PFA versus thermal ablation; heterogeneity was high for several outcomes. |
| **Amin et al., 2024 (16)** | Search through September 2023; 17 studies and 2,255 patients; 1 RCT and 16 nonrandomized studies; paroxysmal and persistent AF; RFA and CBA comparators. | Mixed-design comparative meta-analysis using RR and MD, with RoB 2 and ROBINS-I assessments and leave-one-out sensitivity analyses; no formal GRADE assessment. | PFA was associated with lower AF recurrence, shorter procedure time, fewer phrenic nerve and esophageal injuries, and higher pericardial tamponade. | The dominance of nonrandomized studies, mixed AF phenotypes, and pooled thermal comparators limited causal interpretation and may have influenced rare-event safety estimates. |
| **Esteves et al., 2025 (17)** | Search through February 2025; 1 RCT and 5 observational studies; 3,163 patients undergoing first PVI for paroxysmal AF; PFA compared with RFA. | Mixed-design random-effects synthesis of procedural, 1-year efficacy, and complication outcomes, with RoB 2 and ROBINS-I assessments and leave-one-out analyses; no formal GRADE assessment. | PFA had shorter procedure time and longer fluoroscopy time, with comparable 1-year success and major complication rates. | Only one randomized trial was available; observational confounding and restriction to RFA comparators limited randomized and broader thermal-comparator inference. |
| **Krishan et al., 2025 (18)** | Search through April 2025; 2 RCTs and 817 patients; ADVENT and SINGLE SHOT CHAMPION; first-ablation paroxysmal AF. | RCT-only random-effects synthesis of 12-month clinical and procedural outcomes, with a cryoballoon comparator subgroup; no formal GRADE or leave-one-out analysis. | No statistically detectable clinical differences; procedure and LA dwell times and pooled GA use were lower with PFA. | The two-trial evidence base could not evaluate optimized point-by-point RFA, a distinct nanosecond PFA platform, or the full extent of platform, monitoring, and safety-definition heterogeneity. |
| **Mojica et al., 2026 (19)** | Search through May 2025; 4 RCTs and 1,294 patients; paroxysmal and persistent AF; included a dual-energy PFA/RFA trial and a 24-hour physiology-focused trial. | RCT-only random-effects analysis with GRADE, leave-one-out sensitivity analyses, and meta-regression in a broader AF and intervention framework. | Efficacy and short-term safety were comparable; procedure time was shorter with PFA but heterogeneous; certainty was predominantly low or very low. | Clinical and methodological heterogeneity arose from mixed AF phenotypes, intervention types, and follow-up windows; BEAT PAROX-AF and InsightPFA were not included. |
| **Present review** | Search through March 2026; 4 dedicated RCTs and 1,393 patients undergoing first catheter ablation for paroxysmal AF: ADVENT, SINGLE SHOT CHAMPION, BEAT PAROX-AF, and InsightPFA. | Trial-level endpoint harmonization and exact data mapping; GRADE; leave-one-out analyses for time-based procedural outcomes; dedicated assessment of platforms, comparators, workflows, rhythm monitoring, and serious-adverse-event definitions. | No statistically detectable difference in 12-month efficacy; safety estimates remained imprecise; procedure and LA dwell times favored PFA but were highly heterogeneous and of very low certainty; GA use was interpreted as a contextual process measure. | Extends the randomized evidence base to optimized point-by-point RFA and nanosecond PFA comparisons and provides a more granular interpretation of efficacy, safety, and procedural findings across nonuniform trial configurations. |

Interpretive note: Prior reviews addressed overlapping but nonidentical questions. The present review restricts synthesis to dedicated RCTs of first-ablation paroxysmal AF and explicitly maps trial-level differences affecting pooled interpretation.

Sources: Prior meta-analyses, references 14-19; included randomized trials, references 10-13.

Supplemental Figure S1. Forest plot for leave-one-out sensitivity analysis of total procedure time. Each row shows the pooled mean difference after omitting the named study. Values to the left favor PFA; values to the right favor thermal ablation.

**Abbreviations:** CI = confidence interval; MD = mean difference; PFA = pulsed field ablation.

Supplemental Figure S2. Forest plot for leave-one-out sensitivity analysis of fluoroscopy time. Each row shows the pooled mean difference after omitting the named study. Values to the left favor PFA; values to the right favor thermal ablation.

**Abbreviations:** CI = confidence interval; MD = mean difference; PFA = pulsed field ablation.

Supplemental Figure S3. Forest plot for leave-one-out sensitivity analysis of left atrial dwell time. Each row shows the pooled mean difference after omitting the named study. Values to the left favor PFA; values to the right favor thermal ablation.

**Abbreviations:** CI = confidence interval; LA = left atrial; MD = mean difference; PFA = pulsed field ablation.

Supplemental Figure S4. Risk-of-bias assessment for included randomized controlled trials using the revised Cochrane Risk of Bias 2 tool. Domains are shown as D1 to D5: D1, bias arising from the randomization process; D2, bias due to deviations from intended interventions; D3, bias due to missing outcome data; D4, bias in measurement of the outcome; D5, bias in selection of the reported result. Green circles indicate low risk of bias and yellow circles indicate some concerns.

**Abbreviations:** RoB 2 = Risk of Bias 2.
